# Genome-Wide Analysis of Multiple Organellar RNA Editing Factor Family in Poplar Reveals Evolution and Roles in Drought Stress

**DOI:** 10.3390/ijms20061425

**Published:** 2019-03-21

**Authors:** Dongli Wang, Sen Meng, Wanlong Su, Yu Bao, Yingying Lu, Weilun Yin, Chao Liu, Xinli Xia

**Affiliations:** 1 Beijing Advanced Innovation Center for Tree Breeding by Molecular Design, National Engineering Laboratory for Tree Breeding, College of Biological Sciences and Technology, Beijing Forestry University, Beijing 100083, China; wangdongli1997@163.com (D.W.); mengsen021124@126.com (S.M.); bjfususan@163.com (W.S.); byaq0556@gmail.com (Y.B.); luyingying2018@bjfu.edu.cn (Y.L.); yinwl@bjfu.edu.cn (W.Y.); 2 State Key Laboratory of Tree Genetics and Breeding, Research Institute of Tropical Forestry, Chinese Academy of Forestry, Guangzhou 510520, China

**Keywords:** *Populus trichocarpa*, multiple organellar RNA editing factor, drought stress, RNA editing, genome

## Abstract

Poplar (Populus) is one of the most important woody plants worldwide. Drought, a primary abiotic stress, seriously affects poplar growth and development. Multiple organellar RNA editing factor (*MORF*) genes—pivotal factors in the RNA editosome in *Arabidopsis thaliana*—are indispensable for the regulation of various physiological processes, including organelle C-to-U RNA editing and plasmid development, as well as in the response to stresses. Although the poplar genome sequence has been released, little is known about *MORF* genes in poplar, especially those involved in the response to drought stress at the genome-wide level. In this study, we identified nine *MORF* genes in the Populus genome. Based on the structural features of MORF proteins and the topology of the phylogenetic tree, the *P. trichocarpa* (Ptr) MORF family members were classified into six groups (Groups I–VI). A microsynteny analysis indicated that two (22.2%) *PtrMORF* genes were tandemly duplicated and seven genes (77.8%) were segmentally duplicated. Based on the *d_N_/d_S_* ratios, purifying selection likely played a major role in the evolution of this family and contributed to functional divergence among *PtrMORF* genes. Moreover, analysis of qRT-PCR data revealed that *PtrMORFs* exhibited tissue- and treatment-specific expression patterns. *PtrMORF* genes in all group were involved in the stress response. These results provide a solid foundation for further analyses of the functions and molecular evolution of *MORF* genes in poplar, and, in particular, for improving the drought resistance of poplar by genetics manipulation.

## 1. Introduction

Poplar (Populus) has enormous economic and ecological benefits. It has a relatively small genome (485 ± 10 Mb; 2*n* = 38) and is the model forest species for genomic and genetic studies of woody plants owing to the relative ease of experimental manipulation and range of available genetic tools [1,2]. It is characterized by its height, thickness, and rapid growth, but biotic and abiotic stresses have limited its growth [3,4]. Among these stresses, drought is commonly pervasive with the characteristics of repressing stomata, photosynthesis, respiration, altering gene expression, and reducing biomass [5,6]. Drought is destructive and economically damaging for poplar, with important research and economic values [1,2,7,8]. Accordingly, investigating the stress resistance mechanisms of poplar has research and practical implications.

RNA editing primarily occurs in mitochondria and plastids of land plants and plays an important role in transcript maturation by insertions/deletions and conversion editing [9,10,11]. Conversion editing includes C-to-U, U-to-C, and A-to-I editing. U-to-C and A-to-I editing are likely less than C-to-U, which account for the vast majority of RNA editing events [12,13]. The cis-regulatory region, between nucleotide positions −40 and −5 of upstream of the edited site, interacts with trans-regulatory elements involved in identifying the site and catalyzing the conversion, from cytidine to uridine [14]. The organelle RNA recognition motif-containing protein (ORRM), organelle zinc finger (OZ), protoporphyrinogen IX oxidase (PPO), pentatricopeptide repeat (PPR), and RNA editing factor interacting protein (RIP)/multiple organellar RNA editing factor (MORF) families are key trans-acting elements for RNA editing [11,15,16]. The *ORRM* genes are required for RNA editing; the family contains six genes named *ORRM1* to *ORRM6*. Plastid and mitochondrial editing sites are impaired in almost all *orrm*-mutants [16,17,18,19,20]. OZ1 and PPO1 have also recently been implicated in RNA editing and are all located in plastids [21,22]. PPR proteins include many family members and have been studied extensively [23]. Several PPR functions are in a broad range of events, including developmental and physiological processes and response to various biotic and abiotic stresses [24]. For instance, a PPR96 deficiency in *A. thaliana* is associated with insensitivity to ABA and oxidative stress [23]. Mutations in SLO2 of the E+ subclass of the P-L-S subfamily of PPRs retard leaf emergence, restrict root growth, and result in late flowering, and these parameters are enhanced in the absence of sucrose. Additionally, four RNA editing defects and reduced editing at three sites in *slo2* mutants have been identified [25]. The conversion of nucleotides can not succeed without protein–protein interactions between certain PPRs and MORFs. *(PLS)_3_PPR* and *LPA66*—two PPR genes—are associated with increased RNA-binding activity based on the presence of *MORF9* binding in *A. thaliana* [26]. MORF proteins interact with specific PPR proteins based on pull down in vivo and yeast two-hybrid assays [15].

MORFs are a small protein family in land plants (nine members in *A. thaliana* and seven members in maize) [27,28]. The name of DAG-like (DAL) gene family in maize was previously adopted based on the first identified member (DAG) of the gene family in *Antirrhinum majus* [27,28]. In *A. thaliana*, MORF proteins were also named RNA editing factor interacting proteins (RIP). There are nine members, defined as MORF1–9, while there are ten RIPs. RIPs or MORFs have been used interchangeably, except for RIP1, which corresponds to MORF8, and RIP8, which has been referred to MORF1. Only one gene, *RIP10* (At1g53260), was specially not defined as belonging to the MORF family [9]. In *A. thaliana*, all MORF proteins have no annotated domains but shared a similarly conserved domain. MORF2 and MORF9 are targeted to plastids, and MORF8 is located in chloroplasts and mitochondria, and the others are located in mitochondria. Some *MORF* genes work together and influence each other during some RNA editing events, i.e., they exhibit homo- and heteromeric interactions. For example, both mitochondrial MORF1 and plastid MORF2 proteins can interact with the dual-targeted MORF8 protein. MORF2 and MORF9 are both required for editing at several sites. The presence or absence of MORF8 influences edited sites targeted by MORF2 and/or MORF9 [27,29].

Several members of the RNA editosome interacted with *MORF* family genes towards their important roles in plant growth, development, and RNA editing efficiency [30,31]. Plant development would be negatively affected without MORF proteins. In rice, owing to impaired chloroplast development, the *wsp1* mutant has the variegated phenotype and reduced chlorophyll content. Further, photosynthetic efficiency, CO_2_ gas conductance, and transpiration rate of *wsp1* plants are lower than those of the wild type [32]. In *A. thaliana*, *morf2* and *morf9* mutants show a lack of chlorophyll in leaves, and the T-DNA insertional *rip1* (RIP1 also named as MORF8) mutant demonstrates dwarfism [27,33].

In poplar, functional studies of MORF proteins focused on biotic and abiotic stresses are sparse. In this study, we predicted nine putative *MORF* genes in the *P. trichocarpa* genome. A comprehensive analyses of the poplar MORF family, including phylogenetic, gene structure, chromosomal distribution, and synteny analyses, were performed. The expression profiles of *PtrMORF* genes under drought were determined using public microarray and quantitative RT-PCR data. Our results provide insight into the fascinating properties and biological functions of *MORF* genes in response to drought stress in poplar.

## 2. Results

### 2.1. Identification and Sequence Analysis of the PtrMORF Gene Family

**Figure 1:**
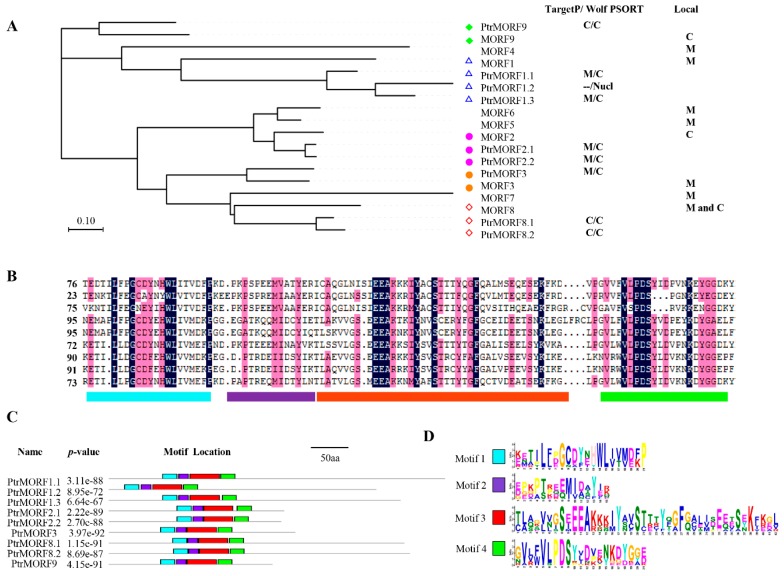
Poplar multiple organellar RNA editing factor (MORF) proteins and their conserved motifs. (**A**) Multiple sequences alignment of *A. thaliana* and *P. trichocarpa* MORF proteins was carried out using MUSCLE, and the Neighbor-Joining (NJ) tree was built using MEGA v7.0. And the chloroplast, mitochondrial or nuclei transit peptides of poplar MORF proteins were predicted using TargetP (http://www.cbs.dtu.dk/services/TargetP/) and Wolf PSORT (http://wolfpsort.org/). M, mitochondria; C, chloroplast; Nucl, nuclei. Green diamond, MORF9 and PtrMORF9; Blue triangle, MORF1, PtrMORF1.1, PtrMORF1.2 and PtrMORF1.3; Red circle, MORF2, PtrMORF2.1 and PtrMORF2.2; Orange circle, MORF3 and PtrMORF3; Orange diamond, MORF8, PtrMORF8.1 and PtrMORF8.2. Local meant the gene where was located in (**B**). Alignment of conserved MORF domains in poplar MORF proteins was conducted using DNAMAN (https://www.lynnon.com/). Lake blue, Motif1; purple, Motif2; red, Motif3; green, Motif4. (**C**,**D**) Putative motifs were explored using the MEME server with the parameters of between 15 and 50 aa in length and sharing of each motif among all PtrMORF proteins.

We searched the poplar genome with known *A. thaliana* MORF proteins as queries. Initially, nine putative *PtrMORF* genes were obtained—*PtrMORF1.1*, *PtrMORF1.2*, *PtrMORF1.3*, *PtrMORF2.1*, *PtrMORF2.2*, *PtrMORF3*, *PtrMORF8.1*, *PtrMORF8.2*, and *PtrMORF9* based on a phylogenetic analysis using the amino acid sequences of all MORFs of *P. trichocarpa* as well as those of *A. thaliana* (Figure 1A). Poplar MORF proteins were predicted using TargetP and Wolf PSORT to enter mitochondria, chloroplasts, or nuclei, like their homologs in *A. thaliana* (Figure 1A). No known motif in poplar MORF proteins was found in the PFAM and INTERPRO databases, but the MORF box was identified, as in previous studies [27,28,33]. Novel putative motifs were explored using the MEME server. By selecting a motif length of between 15 and 50 aa, we identified four conserved motifs located in the MORF domain (Figure 1B–D).

**Figure 2:**
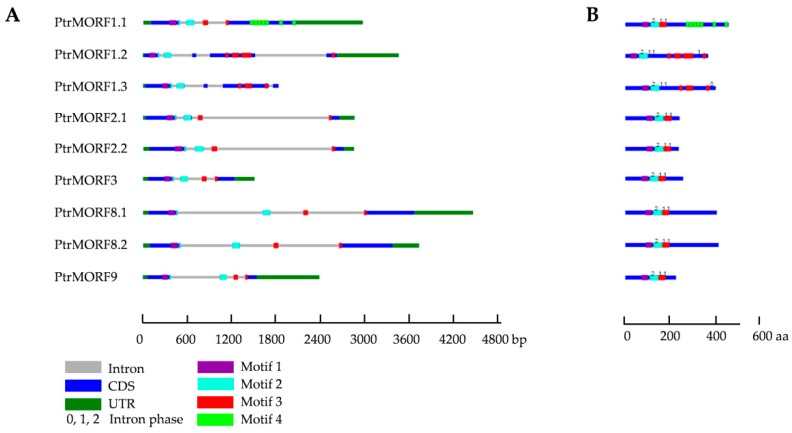
Gene structures of poplar *MORF* genes. (**A**,**B**) The gene structures of *PtrMORF* genes were built using GSDraw (http://wheat.pw.usda.gov/piece/GSDraw.php) by submitting both genomic sequences obtained from PopGenIE (http://popgenie.org/) and coding sequences (CDS) of *PtrMORF* genes.

We further analyzed the sequence structures of the nine *PtrMORF* genes. An alignment of the genomic sequences to predicted CDS sequences of *PtrMORF* genes, showed that *PtrMORF* genes had a conserved gene structure. The *PtrMORF* genes had three introns with intron phases 0, 1, and 2. In all *PtrMORF* genes, motif 1 was encoded by exon 1, motif 2 was encoded by exon1 and exon 2, and motif 3 was encoded by exons 3 and 4, but motif 4 was only located in exon 4 of *PtrMORF1.1* (Figure 2A,B). The nine *PtrMORF* genes ranged from 1515 to 4441 bp and contained four or five exons (Figure 2A). All identified poplar *MORF* genes encoded proteins ranging from 229 to 470 amino acids, and their sequences contained zero to two transmembrane domains (TMDs). The molecular weight (MW) of the nine putative proteins ranged from 26.0 to 51.7 kDa. The GRAVY values of putative MORFs were negative and ranged from −1.382 to −0.602 (Table S1).

### 2.2. Phylogenetic Comparison of the MORF from Different Species

To investigate their molecular evolution and functions in poplar, a phylogenetic analysis of MORF proteins was performed. We used the HMMER 3.0 package to build a Hidden Markov Model (HMM) file (morf.hmm) with 19 MORF domain sequences of MORF proteins from *A. majus*, *P. trichocarpa*, and *A. thaliana* (Table S2). To mine additional MORF domain-encoding genes in other plants, we used the morf.hmm algorithm to query the genomes of six species, representing major evolutionary lineages, including *Arabidopsis lyrata*, *Brachypodium distachyon*, *Glycine max*, *Oryza sativa Japonica*, *Prunus persica*, and *Vitis vinifera*. The numbers of MORF genes in eight species were comparable, ranging from six (in *P. persica*) to 11 (in *A. lyrata* and *G. max*).

**Figure 3:**
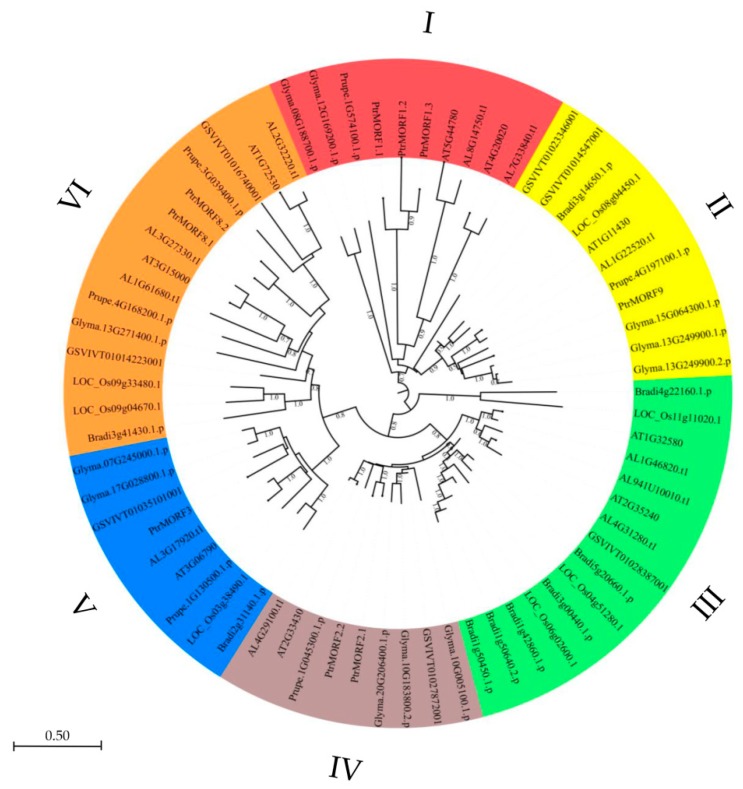
Phylogenetic relationships of the *MORF* gene family members from poplar (*PtrMORFs*) *Arabidopsis lyrata*, *Arabidopsis thaliana*, *Brachypodium distachyon*, *Glycine max*, *Oryza sativa Japonica*, *Prunus persica*, and *Vitis vinifera*. The phylogenetic tree was constructed with MEGA 7.0 (http://www.megasofware.net/mega.html/) program by the neighbor-joining method.

In total, 69 MORF genes were identified in eight plant genomes to build an unrooted tree using MEGA7.0 by employing the neighbor-joining (NJ) method. As shown in Figure 3, sequences were classified into six groups (I, II, III, IV, V, and VI). Each group included *MORF* genes from diverse plant taxa. Among these, classes III and VI were larger than the others, containing 30 members and accounting for 42.5% of all predicted *MORF* genes. *PtrMORF* genes were found in all classes other than group III.

### 2.3. Chromosomal Distribution, Synteny, and Evolution of PtrMORF Genes

**Figure 4:**
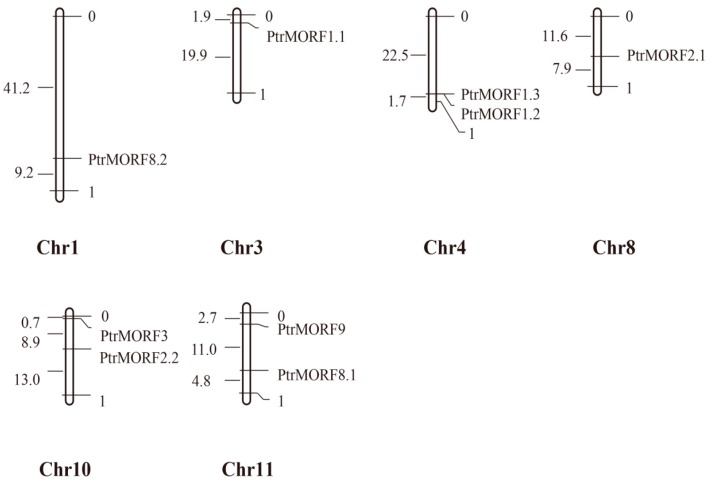
Chromosomal locations of Populus *MORF* family members. The chromosomal locations of the *MORF* genes were mapped with MapDraw program. The number to the left of each chromosome represented the size of the chromosome in Mbp.

**Figure 5:**
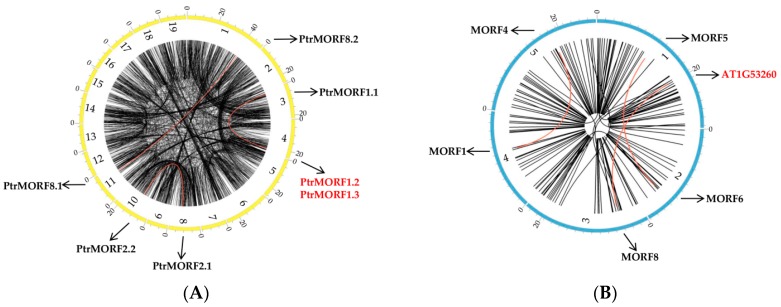
Interchromosomal relationships of *MORF* genes. (**A**) Interchromosomal relationships of *MORF* genes in poplar. (**B**) Interchromosomal relationships of *MORF* genes in *A. thaliana*. Black lines indicated all synteny blocks in the Populus or *A. thaliana* genome and the red indicated synteny blocks where *MORF* duplicated gene pairs were. The chromosome number was indicated at the bottom of each chromosome and each cell on the outside of chromosomes represented 5 Mbp in *Populus* and 1.5 Mbp in *A. thaliana*.

Figure 4 showed that the genes were distributed on six poplar chromosomes, including chromosomes 1, 3, 4, 8, 10, and 11. Half of the chromosomes had two *PtrMORF* members and the other half had a single *MORF*. Tandem duplication was defined as different members of the gene family occurring within the same or neighboring intergenic region [34]. One tandem duplication event involving two *MORF* genes (*PtrMORF1.2*/*PtrMORF1.3*) was identified by BLASTP and MCScanX methods. In addition to the tandem duplication, seven *PtrMORF* genes were assigned to three segmental duplication events (*PtrMORF1.1/PtrMORF1.2/PtrMORF1.3*, *PtrMORF2.1/PtrMORF2.2*, and *PtrMORF8.1/PtrMORF8.2*) in Populus linkage groups 1, 3, 4, 8, 10, and 11. Notably, *PtrMORF1.2* and *PtrMORF1.3* occurred in both tandem and segmental duplications. A cross-matching event was also found in three doubling blocks of *PtrMORF* genes; the chromosomal fragment in which a gene was located was identical to more than one nonself chromosomal segment. *PtrMORF1.1*, *PtrMORF1.2*, and *PtrMORF1.3* synteny blocks corresponded to the third, fourth, and fourth chromosomes, respectively (Figure 5A and Table S3). Figure 5B showed five of the nine *MORF* genes were involved in three segmental duplication events (*MORF1/MORF4*, *MORF5/MORF6*, and *MORF8*/AT1G53260) in *A. thaliana*. It was worth noting that the gene, AT1G53260, was not included in the scope of *MORF* genes because of partial MORF box in our research [27]. The *MORF* gene of *A. thaliana* was also shown to be highly segmental duplicated, which was similar to that in poplar.

**Figure 6:**
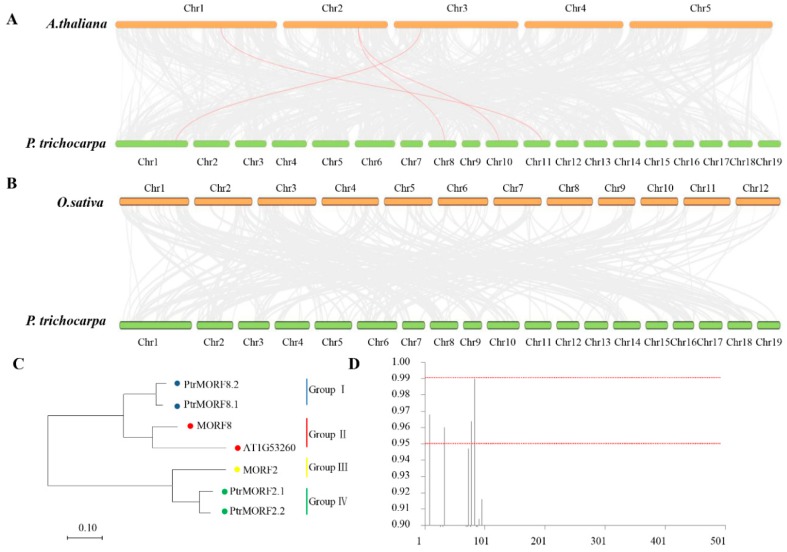
Synteny analysis of MORF genes between poplar and two representative plant species. (**A**,**B**) Gray lines in the background indicate the collinear blocks within poplar and other plant genomes, while the red lines highlight the syntenic MORF gene pairs. (**C**) The phylogenetic relationship of 7 members of four pairs. (**D**) The Bayes Empirical Bayes (BEB) probabilities for sites in the positively selected class (ω > 1). The x-axis denotes position in the amino acid alignment.

We further inferred the phylogenetic relationships of *PtrMORFs*, based on the diverse roles of DYW-PPR protein binding to MORFs on RNA editing sites in *A. thaliana* [9]. We constructed two syntenic maps of poplar with *A. thaliana* and rice. A total of four *PtrMORF* genes showed syntenic relationships with *MORFs* in *A. thaliana* (Figure 6A,C and Table S3). However, no synteny was in rice (Figure 6B). We performed phylogenetic analyses of these MORF proteins in Populus and *A. thaliana*. The ratio of nonsynonymous/synonymous substitution rates of *PtrMORFs* and *MORFs* in *A. thaliana* was determined to evaluate the selection pressure on amino acid substitutions (ω = d_N_/d_S_) and the role of Darwinian positive selection in driving gene divergence after duplication [35,36,37]. Generally, ω > 1 indicates positive selection, ω < 1 provides evidence for negative or purifying selection, and ω = 1 supports neutral evolution.

Using the maximum likelihood method and codon substitution models implemented in PAML, the selection pressure in the four groups of *MORF* genes was evaluated by likelihood ratio tests (LRTs). The estimated ω value for all MORF genes was 0.055 using a one-ratio model (M0). We then detected the positive selection acting on particular groups using a branch model in which each clade had its own ω value. The LRT statistic suggested that the ω values for groups II, III, and IV were significantly different from that of group I, and the ω estimates for all groups other than group I still suggested purifying selection (Table 1).

Thus, we estimated the evolutionary forces acting on individual codon sites using site-specific likelihood models of codon substitution because positive selection was unlikely to affect all sites over prolonged time periods. Three pairs of models—M1 (neutral) and M2 (selection), M0 (one ratio) and M3 (discrete), and M7 (beta) and M8 (beta & ω)—formed three LRTs. As shown in Table 2, model M1 was not significantly worse than M2, although it suggested that 6.6% of sites were nearly neutral with ω = 1. Model M3 with K = 8 suggested that 0.7% of sites were under positive selection, and model M3 was significantly better than the one rate model. Model M8, in which an additional ω ratio was estimated from the data, was not significantly better than M7, indicating that no sites were under positive selection. 

To better detect positive selection, the branch-site model was also applied to evaluate selection on all amino acids of MORF proteins in specific groups (Table 3). LRT showed that model A fitted the data significantly better than the site-specific model M1 (*p* < 0.01) in group II, implying positive selection on 29.7% of sites in group II. At the posterior probability (*p*) > 95%, four sites were likely to be under positive selection in group II. Referring to first sequence in group II, *MORF8*, these positively selected sites were 8T, 33R, 78D, and 83V (Figure 6D). However, no positive selection was found in the branch including *PtrMORFs*.

### 2.4. Expression of PtrMORF Genes and Six Genes from Chloroplasts and Mitochondria under Drought Stress

Although the role of MORF proteins in plastid development and RNA editing in *A. thaliana* and rice have been studied, little is known about how *MORFs* respond to abiotic stimuli, such as drought stress, particularly in poplar [27,32].

**Figure 7:**
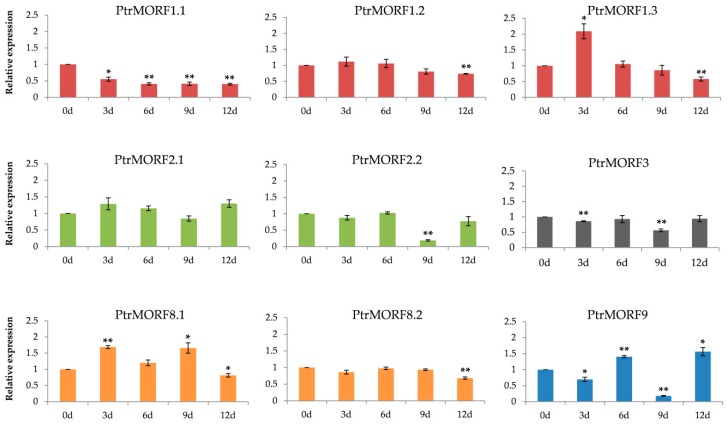
Quantitative RT-PCR analysis of *PtrMORF* genes expression in response to drought stress. 0d, 3d, 6d, 9d, and 12d, drought for 0, 3, 6, 9, and 12 days in a greenhouse environment, respectively. Data were normalized to UBQ gene. The sample without drought treatment was defined as 1 in the figure. The data were presented as the mean ± SE of three separate measurements. Asterisks denote significant differences: * *p* < 0.05; ** *p* < 0.01.

Quantitative real-time reverse transcription-PCR (qRT–PCR) for long-term water deficiency stress in black poplar (Populus × *euramericana cv. ‘Neva’*) was further performed to evaluate the differential expression of each *PtrMORF* gene. Nine genes exhibited significantly different expression under limited water stress except *PtrMORF2.1*, and these could be preliminarily considered as drought-responsive genes. Among them, the two *PtrMORF* genes *PtrMORF1.3* and *PtrMORF8.1* were highly expressed followed by low expression. The expression levels of *PtrMORF1.1* and *PtrMORF1.2*, *PtrMORF2.2*, *PtrMORF3*, and *PtrMORF8.2* under drought conditions were significantly lower than those in control conditions. The mRNA accumulation of *MORF9* fluctuated obviously, exhibiting decreased expression after 3-day and 9-day drought, but increased approximately 1.5-fold after 6-day and 12-day drought. However, the pattern of the response to drought was not consistent. For example, the expression level of *PtrMORF1.1* had downregulated after 3-day drought stress. At the same time, *PtrMORF1.2* and *PtrMORF8.2* were significantly downregulated until 12 days of limited water stress. Furthermore, *PtrMORF* genes that belonged to the same group in the phylogenetic tree had different expression patterns under stress treatment. For instance, *PtrMORF1.1* and *PtrMORF1.3* were downregulated and upregulated in 3-day drought treatments, while *PtrMORF1.2*, also orthologous to group I members and *MORF1* in *A. thaliana*, was not affected by limiting water until 12 days post-treatment (Figure 7).

**Figure 8:**
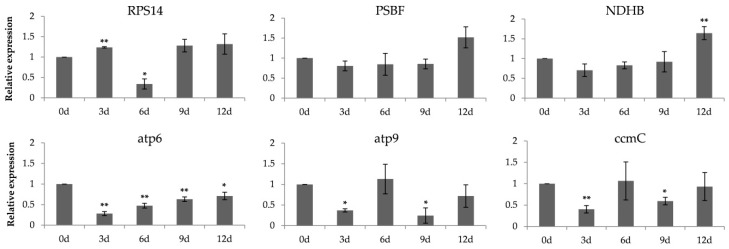
Expressions of three plastid (RPS14, PSBF, and NDHB) and three mitochondrial genes (atp6, atp9, and ccmC) in poplar on the 0, 3rd, 6th, 9th, and twelfth day after drought tolerance. Data were normalized to UBQ gene. The sample without drought treatment was defined as 1 in the figure. The data were presented as the mean ± SE of three separate measurements. Asterisks denote significant differences: * *p* < 0.05; ** *p* < 0.01.

In order to further explore the possible relationship between RNA editing and plant stress, we selected a total of six genes from chloroplasts and mitochondria to evaluate their expression, based on previous studies [27,38,39]. The *RPS14* gene represented important chloroplast proteins, and the fluctuant editing efficiency and gene expression of *RPS14* were associated with cytokinins against stress [39,40]. Under salt stress, *PSBF* and *NDHB* likely showed an elevated editing percentage, linked to PSII repair and increase in *NDHB* gene translation [39]. In rice, *atp6*, *atp9*, and *ccmC* were related to mitochondrial electron transport chain and had been selected to test RNA editing during stress exposure [38]. In our study, these reported genes in poplar were obtained from National Center for Biotechnology information and showed different expression owing to drought. Six genes, except *PSBF*, were significantly upregulated or downregulated after drought stress (Figure 8). The three mitochondrial genes—*atp6*, *atp9*, and *ccmC*—were significantly downregulated after 3-day drought. The level of *RPS14* was highly expressed on 3-day drought followed by a low expression on 6-day drought, and the expression of poplar *NDHB* was significantly increased under 12-day drought.

### 2.5. Expression Profiles of Populus MORF Gene

**Figure 9:**
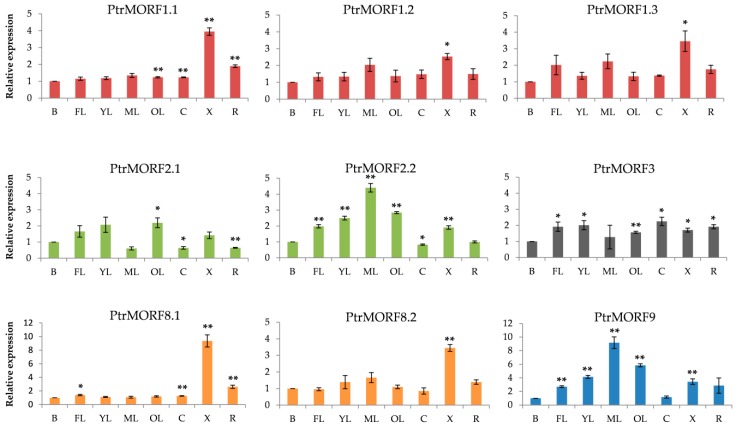
Expression analysis of 9 *MORF* genes in eight representative samples by qRT-PCR. Eight various tissues including buds (B), freshly expanded leaves (FL), expanding young leaves (YL), mature leaves (ML), old leaves (OL), cortex (C), xylem (X), and roots (R). Data were normalized to UBQ gene. The buds were defined as 1 in the figure. The data were presented as the mean ± SE of three separate measurements. Asterisks denote significant differences: * *p* < 0.05; ** *p* < 0.01.

We then examined the tissue-specific expression of nine Populus *MORF* genes by qRT-PCR: we evaluated various tissue types, including buds, freshly expanded leaves, expanding young leaves, mature leaves, old leaves, cortex, xylem, and roots. The expression levels of *PtrMORFs* in these tissues were comparable to those in buds. Some *PtrMORF* genes exhibited clear tissue-specific expression (Figure 9). Five *PtrMORF* genes—*PtrMORF1.1*, *PtrMORF8.1*, *PtrMORF2.2*, *PtrMORF3*, and *PtrMORF9*—were higher or weakly expressed in over four tissues significantly. Among them, the latter three genes were highly expressed level in almost all leaves types, and *PtrMORF8.1* and *PtrMORF1.1* had higher expression levels in freshly expanded leaves and old leaves, respectively. All but one gene—*PtrMORF2.1*—had higher expression in xylem. *PtrMORF1.2*, *PtrMORF1.3*, and *PtrMORF8.2* had no significant difference in their expression in leaves and merely higher expressed in xylem compared to buds. Additionally, The *PtrMORF* genes with closest evolutionary relationship had different expression patterns. *PtrMORF1.1*, *PtrMORF1.2*, and *PtrMORF1.3* had the closest homology relationship with *MORF1* of *A. thaliana.* Among them, *PtrMORF1.1* had high expression levels in old leaves, cortex, xylem, and roots, while *PtrMORF1.2* and *PtrMORF1.3* only in xylem. With respect to *PtrMORF2.1* and *PtrMORF2.2*, which were orthologous to *MORF2* of *A. thaliana*, the latter had high expression level in four leaves types and the former exhibited high expression only in old leaves. A similar inconsistency in expression was observed between *PtrMORF8.1* and *PtrMORF8.2*.

## 3. Discussion

RNA editing plays an irreplaceable role in plant growth and development and C-to-U RNA editing events occur frequently in vascular plants. Trans-acting factors (RNA editosome) are required to recognize nucleotides to be edited, including OZ1, PPR, ORRM, and others. MORF proteins that interact with these factors have been found in *A. thaliana* and *O. sativa* [22,26,32]. *MORF* genes, which are important subunits of the RNA editosome, play a vital role in the regulation of RNA editing [11]. It is worth noting that the functions of most MORF proteins in response to stress, especially drought, remain unclear in woody plant. In this study, we identified the whole *MORF* gene family in poplar and examined the expression patterns of these genes in different plant tissues and in response to drought. The structure of the MORF family, evolutionary events, transcriptional changes responded to drought, and tissue-specific expression pattern are discussed below.

Members of the MORF family have been identified and characterized in many taxa. For example, 10 *MORF* genes have been identified in *A. thaliana*, but At1g53260 (RIP10) may exhibit a partial lack of partial functionality owing to incomplete *MORF* box. We focus on nine *MORF* genes in *A. thaliana* in this study [9,27]. In maize, seven putative the DAG-like (*DAL*) genes have also have been identified. It is worth noting that DAL, MORF, and RIP genes are the same [28]. We screened the *P. trichocarpa* genome for putative *MORF* genes using a tailor-made HMM file derived from multiple MORF domain alignments. We identified nine *PtrMORF* genes consistent with the findings of previous report [28]. Compared with *MORF* genes in *A. thaliana* and in maize (*Zea mays*), the family has not been extensively studied in poplar, indicating that *P. trichocarpa* MORF proteins might perform functions similar to those preformed by their homologs in herbaceous plants.

In our study, to better understand the evolution of the *MORF* gene family in poplar, the structure, conserved motifs, phylogenetic relationships, and collinearity of *PtrMORF* genes were characterized. Four conserved motifs were located in the MORF domain, suggesting that the MORF domain is conserved among *A. thaliana* and *Populus* proteins. Most of the *PtrMORF* genes exhibited similar numbers of exons. A phylogenetic analysis revealed that *PtrMORF* genes and putative *MORF* genes from other species could be classified into six subgroups. The distribution of genes among the subclasses indicated that the expansion of the MORF family occurred before the divergence of the species. Most of *PtrMORF* genes were grouped with *MORF* genes from *A. thaliana* and *P. persica* genomes, indicating a close relationship among *MORF* genes from these species. Gene duplication is a major mechanism underlying the evolution of novel protein functions. We detected 2 (22.2%) *PtrMORF* genes that were tandemly duplicated and seven genes (77.8%) that were segmentally duplicated, implying low tandem and high segmental duplication rates in *PtrMORF* genes. Three homologous pairs of chromosomes included seven of the nine *PtrMORF* genes, with segmental duplication in the poplar genome. For example, homologous chromosomes 8 and 10 both contained one *MORF* gene each; similar findings were obtained for homologous chromosomes 1 and 11, as well as 3 and 4. Two *MORF* genes—Potri.010G007200 and Potri.011G032900—were detected on chromosomes 10 and 11, but their homologous chromosomes 8 and 1 lacked *PtrMORF* genes. Tandem duplication was detected (Table S3). Furthermore, the synteny block including *PtrMORF1.1*, *PtrMORF1.2*, and *PtrMORF1.3* was attributed to multiple copies of the chromosome. These results indicated that some *PtrMORF* genes were possibly generated by gene duplication and segmental duplication events likely served as driving force in *PtrMORF* evolution.

Additionally, synteny maps between two representative species and poplar were constructed to better understand the phylogenetic relationships. Four pairs were detected in *A. thaliana* and none was detected in rice indicating a weak homology relationship between poplar and rice. The ω values for duplicated gene pairs between *PtrMORF* genes and *MORFs* in *A. thaliana* were calculated to explore selective pressures (Table 1, Table 2 and Table 3). In general, the *MORF* genes in the two plants were under strong purifying selection using the branch model. The ω values for four groups were less than 1, except for group II, *MORF8* (At3G15000) and At1G53260 (RIP10), indicating that purifying selective pressure was strong. Positively selected sites might cause adaptive changes after gene duplication and during the evolution of *MORFs* in *A. thaliana*.

RNA editing could potentially contribute to plant resistance to abiotic tolerance on the basis of previous studies [38,41,42]. And some genes closely related to RNA editing, such as *PPR* genes in *A. thaliana* or rice, their mutants changed morphological characteristics due to environmental forces [32,43]. Additionally, *MORF* genes were interacted with *PPR* genes to establish complex editosomes in plant [26,27]. Therefore, we made an attempt to confirm whether the *PtrMORF* genes responded to stress. The eight *PtrMORF* genes distributed in all groups were upregulated or downregulated significantly under drought treatment. It was indicated that they may respond to drought stress. Additionally, the sensitivity of *PtrMORF* genes responding to drought stress was different. The expressions of *PtrMORF1.2*, *PtrMORF2.2*, and *PtrMORF8.2* changed significantly after nine or even 12 days of drought, while the other five genes had lower or higher expression only in three-day drought restriction. This might implied that the former three genes are less sensitive to drought than the latter five. However, not all the genes, which were responded to drought on day three, had consistent response as drought stress increases. This was similar to the response of rice some *PPR* genes to drought stress [44]. Additionally, five of the six genes from poplar chloroplasts and mitochondria showed obviously higher or lower expression compared with no drought treatment, and the edited site efficiency of these genes were affected when the *MORF* genes were mutated out in *A. thaliana* [27], suggesting that the *PtrMORF* gene family, as important component of RNA editing, might be involved in the response to drought stress. However, many questions were worth exploring: (1) How would RNA editing perform under stress? (2) What were the deeper mechanisms of *MORF* genes, as important editosome members of RNA editing?

The functional divergence of *MORF* genes was speculated depended on their tissue-specific expression. Firstly identified member in *A. majus*, the *DAG* gene is essential for chloroplast development in the leaves and etioplast formation of cotyledons [45]. In *A. thaliana*, the *DAG-like* gene is involved in early chloroplast differentiation [46]. Our study showed that six *PtrMORF* genes are expressed highly in the leaves of black poplar (Populus × *euramericana cv. ‘Neva’*), suggesting that *PtrMORF*s might play an important role in chloroplast differentiation and development. Some genes that were highly expressed highly in both the leaves and other tissues, such as the cortex, xylem, and roots, indicating the expanded function of *PtrMORF*s. The expression of three genes, merely higher expressed in xylem compared to buds. There are homologous genes in *A. thaliana*; *MORF1* was located in both mitochondria and *MORF8* in both mitochondria and chloroplasts and possessed an extended C-terminus with unknown function. In previous reports, glycine-rich regions were observed in the C-termini of MORF1 and MORF8 in *A. thaliana* [16]. The genes exhibited glycine-rich regions may play key roles in the biotic and abiotic stresses [47]. In addition, the firstly discovered glycine-rich protein—*GRP-1*—was highly expressed in buds and vascular tissue in the petunia [48]. Taken together, these reports suggested this hypothesis that glycine-rich regions observed in *MORFs* might support them in responding to adversity and be highly expressed in xylem in poplar. Xylem was important for water transportation in plants and *PtrMORF* genes expressed highly in xylem might be closely related to water regulation, making the *MORF* genes likely respond to drought [49]. However, we had to point out that the exact roles within *PtrMORFs* need further experiments. Additionally, there were still living parenchyma cells in the xylem and the activity of parenchyma cells provided a critical metabolic and energetic role in woody stem [49]. They played a major role in editing implying that the three *MORF* genes in poplar may play unlikely similar roles in annual plants and were probably required in other critical functions in perennial woody plants.

Therefore, this research preliminarily provided insight into the roles of *PtrMORF*s in stress response and investigations of functions for these genes required further experimental validation in future studies.

## 4. Materials and Methods

### 4.1. Plant Materials and Treatments

Black poplar (Populus × *euramericana cv. ‘Neva’*) were grown in a greenhouse at 25 °C under a 16/8 h light/dark cycle. The 3.5-month-old plants were subjected to drought stress. Twelve plants were encountered water-limited treatment, ranging from 3 to 12 days of drought in which the soil RWC was reduced from 70%; the other three plants were provided abundant water, with a the soil RWC of greater than 30% [50]. Three biological replicates were performed. 

Total RNA was isolated from poplar mature leaves (sixth to twelfth) after drought stress. Different organs and tissues of 3.5-month-old plants, including buds, freshly expanded leaves (second to third), expanding young leaves (fourth to fifth), mature leaves (sixth to twelfth), old leaves (leaves below the mature leaves), cortex, xylem (the stem without cortex), and roots, were collected at the same time and immediately immersed in liquid nitrogen. 

### 4.2. Genome-Wide Identification and Sequence Analysis of MORF Genes in P. trichocarpa

The potential *MORF* genes in *P. trichocarpa* were queried using a local BLASTP search with an E-value threshold of <10^−10^ and a bit score of >100 in poplar genome annotation data (Ptrichocarpa_210_v3.0.protein.fa.gz downloaded from https://phytozome.jgi.doe.gov/pz/portal.html) based on preexisting MORF/RIP genes, including At1g11430, At1g32580, At1g72530, At2g33430, At2g35240, At3g06790, At3g15000, At4g20020, and At5g44780 from *A. thaliana*, which was then confirmed in previous studies [27,28]. Multiple sequence alignments of PtrMORF proteins and the known MORF proteins were generated using DNAMAN. The alignment results were used to build protein Hidden Markov Models (HMMs) to mine the conserved domain; the file was named morf.hmm and was generated using the hmmbuild program in the HMMER 3.0 package (version 3.1b2). No known motifs in PtrMORF proteins and MORF proteins of other plants were detected by screening the PFAM (http://pfam.sanger.ac.uk/) and INTERPRO (http://www.ebi.ac.uk/interpro/) databases [51,52]. The HMM file was used as a probe to search genome files of representative species downloaded from Phytozome V11.0, including *A. lyrata*, *B. distachyon*, *G. max*, *O. sativa Japonica*, *P. persica*, and *V. vinifera* [53]. Protein hits with an E-value of <10^−10^ and sequence score of “best 1 domain” >100 were collected.

MEME (http://meme.nbcr.net/meme/cgi-bin/meme.cgi) was used to investigate the putative conserved motifs among PtrMORF proteins with the following parameters: length between 15 and 50 aa, maximum number of motifs = 4, and one per sequence. To obtain the intact conserved MORF domain, different limits for the length of each motif were used between 100 and 120 aa [54]. In addition, TargetP, and Wolf PSORT were used to predict the putative organelle localization of PtrMORF proteins [55,56].

### 4.3. Phylogenetic Analysis

A multiple sequence alignment of 69 MORF proteins from *P. trichocarpa* and other species including *A. lyrata*, *A. thaliana*, *B. distachyon*, *G. max*, *O. sativa Japonica*, *P. persica*, and *V. vinifera* was generated using the MUSCLE method. A phylogenetic tree was constructed by using the NJ method implemented in MEGA V7.0 [57]. The parameters for tree construction were as follows. Phylogeny test and options: bootstrap (1000 replicates); gaps/missing data: pairwise deletion; model: Dayhoff model; pattern among lineages: same (homogeneous); and rates among sites: uniform rates. Finally, the phylogenetic tree was visualized using itol (http://itol.embl.de/) [58].

### 4.4. Chromosome Location and Gene Structure Analysis

Positional information and gene structures of *PtrMORF* genes on chromosomes of *P. trichocarpa* were obtained from the PopGenIE database [59]. The chromosomal locations were displayed with MapDraw program [60]. The numbers and organization of introns and exons and gene structures were drawn and displayed using the online PIECE2 server of GSDraw [61].

### 4.5. Colinearity of PtrMORF Gene

The chromosomal locations of *PtrMORF* genes were obtained from PopGenIE. Multiple Collinearity Scan Toolkit (MCScanX) was used to analyze gene duplication events with the default parameters and the colinearity information was showed by Circos [62,63]. Using Dual Systeny Plotter software (https://github.com/CJ-Chen/TBtools), the synteny relationships of orthologous *MORF* genes among poplar, *A. thaliana*, and rice were evaluated [64]. Nonsynonymous (*d_N_*) and synonymous (*d_S_*) substitutions, as well as *d_N_/d_S_* ratio (ω) for duplicated *MORF* genes, were calculated, using branch-specific, site-specific (one, neutral, selection, discrete, beta, and beta & ω > 1), and branch-site (Model A) of codon substitution models, as implemented in PAML version 4 [65,66,67]. Likelihood ratio tests (LRTs) were used to compare model fits. The sites under positive selection were identified using Bayesian methods [68].

### 4.6. RT-PCR and qRT-PCR Analyses

Total RNA was extracted using a Plant RNA EASYspin Plus Kit (Aidlab, Beijing, China) and first-strand cDNA synthesis was performed using approximately 2 μg of RNA using the FastQuant RT Kit (with gDNase) (TIANGEN, Beijing, China) following the manufacturer’s protocols. qRT-PCR was conducted on the ABI StepOnePlus Real-Time PCR System (ABI, Foster City, CA, USA) based on the SYBR Green II method. Twenty microliters of cDNA was diluted 1:10 with nuclease-free water. Each reaction contained 10 µL of SYBR Green qPCR Mix (Aidlab), 0.2 µL of ROX Reference Dye (Aidlab), 1 µL of cDNA (corresponding to 10 ng of total RNA), 7.8 µL of nuclease-free water, and 0.25 µM each primer. The thermal profile for qRT-PCR: 3 min at 94 °C, 40 cycles of 10 s at 94 °C, 20 s at 60 °C, and 72 °C for 30 s. Primers were designed using Primer3 (http://bioinfo.ut.ee/primer3-0.4.0/) [69]. A list of primers used was given (Table S4). Each experiment was performed in three biological replicates. The poplar housekeeping gene, UBQ, was used as an internal control. Relative expression was calculated by the 2^−ΔΔCt^ method [70]. A Student’s *t*-test was used to generate every *p*-value for statistical analyses, and R project was used to identify significant variance (R version 3.5.1, one sample and two sample *t*-test: * *p* < 0.05; ** *p* < 0.01).

## 5. Conclusions

Genome-wide identification and evolutionary, gene structure, and expression analyses under drought stress of poplar *MORF* genes provide a deep insight into the gene family. Moreover, analyses of *MORF* genes based on qRT-PCR validation of poplar tissues provide functional information. Our results provide an important resource for advancing our understanding of the roles of *MORF* genes in poplar.

## Figures and Tables

**Table 1:** Parameter estimates and likelihood ratio tests for the branch model.

Model	P ^a^	LnL ^b^	Estimates of Parameters	ΔLRT ^c^	df	*p*	Positively Selected Sites
M0 (one ratio model)	1	−1366.204	ω = 0.055	-	-	-	None
Branch-specific model (Model 2: two ratios)							
Estimate ω for I	2	−1366.136	ω_I1_ = 0.067, ω_0_ = 0.051	Model 2 vs. M0: 0.136	1	0.712	-
Estimate ω for II	2	−1357.726	ω_II1_ = 0.177, ω_0_ = 0.028	Model 2 vs. M0: 16.956	1	0.000	-
Estimate ω for III	2	−1361.616	ω_III1_ = 0.018, ω_0_ = 0.079	Model 2 vs. M0: 9.176	1	0.002	-
Estimate ω for IV	2	−1363.519	ω_IV1_ = 0.010, ω_0_ = 0.083	Model 2 vs. M0: 5.370	1	0.002	-

a: The number of free parameters for the ω ratios; b: lnL value; c: likelihood ratio test.

**Table 2:** Parameter estimates and likelihood ratio tests for the site model.

Model	P ^a^	LnL ^b^	Estimates of Parameters	ΔLRT ^c^	df	*p*	Positively Selected Sites
M0 (one ratio model)	1	−1366.204	ω = 0.0546	-	-	-	None
Site-specific models							
M1 (K = 2)	1	−1363.770	*p*0 = 0.926, (*p*1 = 0.074)	-	-	-	Not allowed
M2 (K = 3)	4	−1363.770	*p*0 = 0.926, *p*1 = 0.008, (*p*2 = 0.066), ω2 = 1.000	M2 vs. M1: 0.000	2	1.000	None
M3 (K = 8)	5	−1346.619	*p*0 = 0.485, *p*1 = 0.001, *p*2 = 0.192, *p*3 = 0.174, *p*4 = 0.029, *p*5 = 0.040, *p*6 = 0.074, *p*7 = 0.007; ω0 = 0.006, ω1 = 0.062, ω2 = 0.062, ω3 = 0.062, ω4 = 0.266, ω5 = 0.266, ω6 = 0.266, ω7 = 0.266	M3 vs. M0: 39.170	14	0.000	None
M7	2	−1347.154	*p* = 0.441, q = 6.096	-	-	-	Not allowed
M8	4	−1347.154	*p*0 = 0.999, *p* = 0.441, q = 6.096, (*p*1 = 0.000), ω = 1.000	M8 vs. M7: 0.000	2	1.000	None

a: The number of free parameters for the ω ratios; b: lnL value; c: likelihood ratio test.

**Table 3:** Parameter estimates and likelihood ratio tests for the branch-site model.

Model	P ^a^	LnL ^b^	Estimates of Parameters	ΔLRT ^c^	df	*p*	Positively Selected Sites
M1: Neutral	1	−1363.770	*p*0 = 0.926, (*p*1 = 0.074)	-	-	-	-
Branch-site models							
Model A (I)	3	−1361.803	*p*0 = 0.929, *p*1 = 0.000, (*p*2a + *p*2b = 0.071), ω2 = 1.706	Model A vs M1: 3.934	2	1.40 × 10^−1^	-
Model A (II)	3	−1346.578	*p*0 = 0.684, *p*1 = 0.018, (*p*2a + *p*2b = 0.297), ω2 = 1.053	Model A vs M1: 34.384	2	3.40 × 10^−8^	Site for foreground lineage: 4 (at *p* > 0.95)
Model A (III)	3	−1363.521	*p*0 = 0.927, *p*1 = 0.057, (*p*2a + *p*2b = 0.015), ω2 = 17.935	Model A vs M1: 0.500	2	7.78 × 10^−1^	-
Model A (IV)	3	−1363.522	*p*0 = 0.925, *p*1 = 0.064, (*p*2a + *p*2b = 0.012), ω2 = 2.630	Model A vs M1: 0.496	2	7.81 × 10^−1^	-

a: The number of free parameters for the ω ratios; b: lnL value; c: likelihood ratio test.

## Supplementary Materials

Supplementary materials can be found at https://www.mdpi.com/1422-0067/20/6/1425/s1.
