# Supplementary material for: Genome-Wide Analysis of Multiple Organellar RNA Editing Factor Family in Poplar Reveals Evolution and Roles in Drought Stress
Source: Int J Mol Sci. 2019 Mar 21;20(6):1425. doi: 10.3390/ijms20061425 (PMC6471648; doi:10.3390/ijms20061425)
Supplement: Supplementary file 1 [file ijms-20-01425-s001.zip › ijms-441665 suppl/Table S2.doc]

\# STOCKHOLM 1.0

DAG/MORF	ETIMLPGCDYNHWLIVMEFPKD-PAPTREQMIDTYLNTLATVLGS-MEEAKKNMYAFSTT

Potri.008g169900 MAPLFPGCDYEHWLIVMDKPGG-EGATKQQMIDCYIETLAKVVGS-EEEAKTKIYNVSCE

Potri.010g068300 MAPLFPGCDYEHWLIVMDKPGG-EGATKQQMIDCYIQTLSKVVGS-EEEAKNKIYNVSCE

Potri.004g218600 NTILFEGNEYIHWLVTVDFPKE-PKPSPEEMVAAFERICAQGLNISIEEAKKRMYACSTT

Potri.004g218800 NKTLFEGCAYNYWLVTVDFPKEEPKPSPREMIAAYERICAQGLNSSIEEAKKRIYACSTT

Potri.003g015100 DTILFPGCDYNHWLITVDFPKD-PKPSPEEMVATYERICAQGLNISIEEAKKKIYACSTT

Potri.011g112200 ETILLDGCDFEHWLVVMDKPEG-D-PTRDEIIDSYIKTLAEVVGS-EEEARKKIYSVSTR

Potri.001g393400 ETILLDGCDFEHWLVVMEKPEG-D-PTRDEIIDSYIKTLAQVVGS-EEEARRKIYSVSTR

Potri.011g032900 ETILLPGCDYNHWLIVMEFPKD-PAPTREQMIDTYLNTLATVLGS-MEEAKKNMYAFSTT

Potri.010g007200 ETILLDGCDYNHWLIVMEFPND-PKPTEEEMINAYVKTLSSVLGS-EEEAKKSIYSVSTT

AT5G44780	DN---EGCDFNHWLITMNFPKDNLP-SREEMISIFEQTCAKGLAISLEEAKKKIYAICTT

AT1G72530	VPSLVEGCDYKHWLVLMKPPNG-Y-PTRNHIVQSFVETLAMALGS-EEEAKRSIYSVSTK

AT4G20020	DTVLFEGCDYNHWLITMDFSKEETPKSPEEMVAAYEETCAQGLGISVEEAKQRMYACSTT

AT2G35240	MAPLFPGCDYEHWLIVMEKPGG-ENAQKQQMIDCYVQTLAKIVGS-EEEARKKIYNVSCE

AT1G32580	MAPLFPGCDYEHWLIVMDKPGG-ENATKQQMIDCYVQTLAKIIGS-EEEAKKKIYNVSCE

AT2G33430	MAPLFPGCDYEHWLIVMDKPGG-EGATKQQMIDCYIQTLAKVVGS-EEEAKKRIYNVSCE

AT3G15000	ETILLDGCDFEHWLVVVEPPQG-E-PTRDEIIDSYIKTLAQIVGS-EDEARMKIYSVSTR

AT3G06790	ETILLDGCDYEHWLIVMEFT-D-PKPTEEEMINSYVKTLTSVLGW-QEEAKKKIYSVCTS

AT1G11430	ETIMLPGCDYNHWLIVMEFPKD-PAPSRDQMIDTYLNTLATVLGS-MEEAKKNMYAFSTT

DAG/MORF	TYTGFQCTVTEETSEKFKG----LPGVLWVLPDSYIDVKNKDYGGDKYVNGEIIP

Potri.008g169900 RYFGFGCEIDEETSNKLEGLFRCLPGVLFVLPDSYVDPEYKDYGAELFVNGEIVQ

Potri.010g068300 RYFGFGCEIDEETSNKLEG----LPGVLFVLPDSYVDPEYKDYGAELFVNGEIVQ

Potri.004g218600 IYQGFQVSITHQEAEKFRGR--CVPGAVFVSPDS---RVKKENGGDKYKNAVITP

Potri.004g218800 TFQGFQVLMTEQESEKFRD----VPRVVFVLPDS---PGNKEYEGDEYEHRMITP

Potri.003 g015100 TYQGFQALMSEQESEKFKD----VPGVVFVLPDSYIDPVNKEYGGDKYENGVITP

Potri.011g112200 CYFAFGALVSEEVSYKIKE----LKNVRWVLPDSYLDVKNKDYGGEPFIDGKAVP

Potri.001g393400 CYYAFGALVPEEVSYKIKE----LKNVRWVLPDSYLDVKNKDYGGEPFIDGKAVP

Potri.011g032900 TYTGFQCTVDEATSEKFKG----LPGVLWVLPDSYIDVKNKDYGGDKYVNGEIIP

Potri.010g007200 TYTGFGALISEELSYKVKA----LPGVLWVLPDSYLDVPNKDYGGDLYEDGKVIH

AT5G44780	SYQGFQATMTIGEVEKFRD----LPGVQYIIPDSYIDVENKVYGGDKYENGVITP

AT1G72530	YYYAFGCRIHEPLTYKIRS----LPDVKWVLPDSFIVDGDNRYGGEPFVDGEVVP

AT4G20020	TYQGFQAIMTEQESEKFKD----LPGVVFILPDSYIDPQNKEYGGDKYENGVITH

AT2G35240	RYFGFGCEIDEETSNKLEG----LPGVLFVLPDSYVDPEFKDYGAELFVNGEVVP

AT1G32580	RYFGFGCEIDEETSNKLEG----LPGVLFILPDSYVDQENKDYGAELFVNGEIVQ

AT2G33430	RYLGFGCEIDEETSTKLEG----LPGVLFVLPDSYVDPENKDYGAELFVNGEIVQ

AT3G15000	CYYAFGALVSEDLSHKLKE----LSNVRWVLPDSYLDVRNKDYGGEPFIDGKAVP

AT3G06790	TYTGFGALISEELSCKVKA----LPGVLWVLPDSYLDVPNKDYGGDLYVEGKVIP

AT1G11430	TYTGFQCTIDEETSEKFKG----LPGVLWVLPDSYIDVKNKDYGGDKYINGEIIP

//

HMMER3/f [3.1b2 | February 2015]

NAME morf

LENG 109

ALPH amino

RF	no

MM	no

CONS yes

CS	no

MAP	yes

DATE Sat Mar 24 22:26:07 2018

NSEQ 19

EFFN 0.765381

CKSUM 460864455

STATS LOCAL MSV	-9.6640 0.71558

STATS LOCAL VITERBI -10.4000 0.71558

STATS LOCAL FORWARD	-4.1265 0.71558

HMM	A	C	D	E	F	G	H	I	K	L	M	N	P	Q	R	S	T	V	W	Y

m->m	m->i	m->d	i->m	i->i	d->m	d->d

COMPO	2.59311 4.16006 2.88692 2.57333 3.20643 2.93547 3.78222 2.77590 2.65519 2.52436 3.63165 3.12908 3.37994 3.13796 3.12672 2.69319 2.86879 2.57205 4.38000 3.13129

2.68618 4.42225 2.77519 2.73123 3.46354 2.40513 3.72494 3.29354 2.67741 2.69355 4.24690 2.90347 2.73739 3.18146 2.89801 2.37887 2.77519 2.98518 4.58477 3.61503

0.02614 4.05309 4.77543 0.61958 0.77255 0.00000	*

1	2.61362 4.90174 2.52289 1.99335 4.16510 3.38040 3.61352 3.58950 2.40771 3.17161 3.10173 2.58029 3.79568 2.75119 2.88332 2.61962 2.84922 2.98170 5.39074 4.03039	1 e - - -

2.68618 4.42225 2.77519 2.73123 3.46354 2.40513 3.72494 3.29354 2.67741 2.69355 4.24690 2.90347 2.73739 3.18146 2.89801 2.37887 2.77519 2.98518 4.58477 3.61503

0.02614 4.05309 4.77543 0.61958 0.77255 0.48576 0.95510

2	2.16677 4.59315 3.03739 2.58128 4.03293 3.30770 3.75578 3.42808 2.42814 3.06828 3.90298 2.81136 3.27822 2.94184 2.99502 2.61751 1.93223 3.08565 5.33182 4.03474	2 t - - -

2.68618 4.42225 2.77519 2.73123 3.46354 2.40513 3.72494 3.29354 2.67741 2.69355 4.24690 2.90347 2.73739 3.18146 2.89801 2.37887 2.77519 2.98518 4.58477 3.61503

0.06453 4.05309 3.09825 0.61958 0.77255 0.48576 0.95510

3	2.53065 4.23447 3.58334 3.05458 3.49479 3.47711 3.94265 1.91384 2.98431 2.51320 3.45113 3.39245 2.91407 3.29492 3.30341 2.56203 2.62814 2.32235 4.97507 3.75048	3 i - - -

2.68618 4.42225 2.77519 2.73123 3.46354 2.40513 3.72494 3.29354 2.67741 2.69355 4.24690 2.90347 2.73739 3.18146 2.89801 2.37887 2.77519 2.98518 4.58477 3.61503

0.02715 4.01570 4.73805 0.61958 0.77255 0.52512 0.89523

4	3.30169 4.69054 4.66044 4.13821 3.07395 4.44338 4.74285 2.21371 3.90494 0.83428 2.64117 4.42502 4.68553 4.14587 4.06579 3.82091 3.54005 2.32921 5.13632 3.98348	4 l - - -

2.68618 4.42225 2.77519 2.73123 3.46354 2.40513 3.72494 3.29354 2.67741 2.69355 4.24690 2.90347 2.73739 3.18146 2.89801 2.37887 2.77519 2.98518 4.58477 3.61503

0.02715 4.01570 4.73805 0.61958 0.77255 0.52512 0.89523

5	3.16174 4.53244 4.63585 4.10078 1.79718 4.31454 4.41057 2.26209 3.97001 1.28893 2.99514 4.28724 4.57666 4.09894 4.10472 3.65106 3.39310 2.11216 4.74425 3.37010	5 l - - -

2.68618 4.42225 2.77519 2.73123 3.46354 2.40513 3.72494 3.29354 2.67741 2.69355 4.24690 2.90347 2.73739 3.18146 2.89801 2.37887 2.77519 2.98518 4.58477 3.61503

0.02715 4.01570 4.73805 0.61958 0.77255 0.46192 0.99446

6	2.83101 5.32348 1.94749 1.65564 4.63392 3.28216 3.71980 4.12289 2.63342 3.63480 4.44734 2.75638 2.36693 2.86683 3.17819 2.75551 3.09935 3.70900 5.80124 4.34833	6 e - - -

2.68618 4.42225 2.77519 2.73123 3.46354 2.40513 3.72494 3.29354 2.67741 2.69355 4.24690 2.90347 2.73739 3.18146 2.89801 2.37887 2.77519 2.98518 4.58477 3.61503

0.02614 4.05309 4.77543 0.61958 0.77255 0.48576 0.95510

7	2.90491 4.65880 3.61539 3.53954 4.67163 0.49785 4.64544 4.31846 3.74517 3.94885 4.92856 3.78019 4.05552 4.05531 3.95816 3.08257 3.40022 3.81190 5.71565 4.78082	7 G - - -

2.68618 4.42225 2.77519 2.73123 3.46354 2.40513 3.72494 3.29354 2.67741 2.69355 4.24690 2.90347 2.73739 3.18146 2.89801 2.37887 2.77519 2.98518 4.58477 3.61503

0.02614 4.05309 4.77543 0.61958 0.77255 0.48576 0.95510

8	2.40817 1.20001 3.82482 3.50198 3.85937 3.15792 4.30168 3.19766 3.35336 3.05298 3.99663 2.87544 3.86830 3.71412 3.56570 2.63879 2.86458 2.85368 5.31150 4.05441	8 c - - -

2.68618 4.42225 2.77519 2.73123 3.46354 2.40513 3.72494 3.29354 2.67741 2.69355 4.24690 2.90347 2.73739 3.18146 2.89801 2.37887 2.77519 2.98518 4.58477 3.61503

0.02614 4.05309 4.77543 0.61958 0.77255 0.48576 0.95510

9	2.64438 5.43669 1.18253 1.98218 4.76303 3.24639 3.80233 4.23320 2.81872 3.78157 4.64072 2.73006 3.86910 2.97256 3.40455 2.84217 3.23515 3.82308 5.96697 4.47709	9 d - - -

2.68618 4.42225 2.77519 2.73123 3.46354 2.40513 3.72494 3.29354 2.67741 2.69355 4.24690 2.90347 2.73739 3.18146 2.89801 2.37887 2.77519 2.98518 4.58477 3.61503

0.02614 4.05309 4.77543 0.61958 0.77255 0.48576 0.95510

10	3.66897 4.91626 4.60669 4.29528 1.56254 4.40105 3.49107 3.37214 4.14454 2.70266 3.97266 4.07649 4.71595 4.13113 4.19323 3.77396 3.89344 3.29525 3.63491 0.93818	10 y - - -

2.68618 4.42225 2.77519 2.73123 3.46354 2.40513 3.72494 3.29354 2.67741 2.69355 4.24690 2.90347 2.73739 3.18146 2.89801 2.37887 2.77519 2.98518 4.58477 3.61503

0.02614 4.05309 4.77543 0.61958 0.77255 0.48576 0.95510

11	2.69576 5.02815 2.72322 1.85266 4.31510 3.37728 3.63474 3.30566 2.24694 3.30413 4.10918 2.16305 3.82350 2.77136 2.83497 2.67971 2.93342 3.38581 5.49314 4.12622	11 e - - -

2.68618 4.42225 2.77519 2.73123 3.46354 2.40513 3.72494 3.29354 2.67741 2.69355 4.24690 2.90347 2.73739 3.18146 2.89801 2.37887 2.77519 2.98518 4.58477 3.61503

0.02614 4.05309 4.77543 0.61958 0.77255 0.48576 0.95510

12	3.07451 4.89322 3.36272 2.99893 2.70137 3.73775 1.34000 3.54522 2.80601 3.01889 4.06205 3.37051 4.17805 3.26684 3.09427 3.12960 3.31969 3.32370 4.29652 2.39694	12 h - - -

2.68618 4.42225 2.77519 2.73123 3.46354 2.40513 3.72494 3.29354 2.67741 2.69355 4.24690 2.90347 2.73739 3.18146 2.89801 2.37887 2.77519 2.98518 4.58477 3.61503

0.02614 4.05309 4.77543 0.61958 0.77255 0.48576 0.95510

13	3.65286 4.96017 4.37839 4.13164 2.87424 3.94187 4.14017 3.68794 3.85192 3.05007 4.31278 4.24515 4.47538 4.23013 3.94728 3.85470 3.96320 3.58317 0.58829 2.86747	13 W - - -

2.68618 4.42225 2.77519 2.73123 3.46354 2.40513 3.72494 3.29354 2.67741 2.69355 4.24690 2.90347 2.73739 3.18146 2.89801 2.37887 2.77519 2.98518 4.58477 3.61503

0.02614 4.05309 4.77543 0.61958 0.77255 0.48576 0.95510

14	3.36335 4.77411 4.47878 4.12301 3.21370 4.20969 4.72237 2.43126 3.88901 0.68095 3.18659 4.38092 4.61626 4.21383 4.03408 3.80702 3.65065 2.51482 5.15480 3.91863	14 L - - -

2.68618 4.42225 2.77519 2.73123 3.46354 2.40513 3.72494 3.29354 2.67741 2.69355 4.24690 2.90347 2.73739 3.18146 2.89801 2.37887 2.77519 2.98518 4.58477 3.61503

0.02614 4.05309 4.77543 0.61958 0.77255 0.48576 0.95510

15	3.13096 4.43655 4.86520 4.37359 3.59380 4.49287 5.08324 1.18498 4.26164 2.08518 3.39439 4.58552 4.80083 4.52282 4.45435 3.88647 3.39783 1.23675 5.57667 4.37335	15 i - - -

2.68618 4.42225 2.77519 2.73123 3.46354 2.40513 3.72494 3.29354 2.67741 2.69355 4.24690 2.90347 2.73739 3.18146 2.89801 2.37887 2.77519 2.98518 4.58477 3.61503

0.02614 4.05309 4.77543 0.61958 0.77255 0.48576 0.95510

16	2.75383 4.30919 4.07083 3.56070 3.55645 3.80008 4.37071 2.12906 3.44330 2.14245 3.45103 3.83081 4.25754 3.76007 3.71018 3.15108 2.16816 1.38787 5.23048 4.01213	16 v - - -

2.68618 4.42225 2.77519 2.73123 3.46354 2.40513 3.72494 3.29354 2.67741 2.69355 4.24690 2.90347 2.73739 3.18146 2.89801 2.37887 2.77519 2.98518 4.58477 3.61503

0.02614 4.05309 4.77543 0.61958 0.77255 0.48576 0.95510

17	3.01408 4.46815 4.43325 3.91333 3.27430 4.12816 4.59017 2.14674 3.74847 1.78713 1.60281 4.16501 4.48812 4.01219 3.94780 3.48863 3.28238 1.72427 5.17636 4.01178	17 m - - -

2.68618 4.42225 2.77519 2.73123 3.46354 2.40513 3.72494 3.29354 2.67741 2.69355 4.24690 2.90347 2.73739 3.18146 2.89801 2.37887 2.77519 2.98518 4.58477 3.61503

0.02614 4.05309 4.77543 0.61958 0.77255 0.48576 0.95510

18	2.89336 5.47401 1.57869 1.66948 4.76516 3.26954 3.73157 4.27718 2.41611 3.75728 4.57269 2.50477 3.84617 2.87936 3.23338 2.79020 3.16312 3.84346 5.90894 4.42190	18 d - - -

2.68618 4.42225 2.77519 2.73123 3.46354 2.40513 3.72494 3.29354 2.67741 2.69355 4.24690 2.90347 2.73739 3.18146 2.89801 2.37887 2.77519 2.98518 4.58477 3.61503

0.02614 4.05309 4.77543 0.61958 0.77255 0.48576 0.95510

19	2.65132 4.50418 3.27301 2.76980 2.17336 3.51445 3.75794 3.04180 2.13908 2.69943 3.62181 3.20334 3.14800 3.05013 2.99894 2.80022 2.89701 2.80574 4.95011 3.60649	19 k - - -

2.68618 4.42225 2.77519 2.73123 3.46354 2.40513 3.72494 3.29354 2.67741 2.69355 4.24690 2.90347 2.73739 3.18146 2.89801 2.37887 2.77519 2.98518 4.58477 3.61503

0.02614 4.05309 4.77543 0.61958 0.77255 0.48576 0.95510

20	2.36293 4.32082 3.22900 2.99002 4.27753 3.05430 4.14255 3.66227 3.04795 3.37039 4.24087 3.25404 1.30996 3.39913 3.36713 2.34130 2.60557 3.19050 5.62208 4.36658	20 p - - -

2.68618 4.42225 2.77519 2.73123 3.46354 2.40513 3.72494 3.29354 2.67741 2.69355 4.24690 2.90347 2.73739 3.18146 2.89801 2.37887 2.77519 2.98518 4.58477 3.61503

0.05200 4.05309 3.40213 0.61958 0.77255 0.48576 0.95510

21	2.74561 5.12575 2.68708 2.18991 4.45371 2.67338 3.62727 3.89847 1.76834 3.40897 4.20877 2.53747 3.83425 2.62698 2.73463 2.71087 2.98041 3.51552 5.56024 4.19681	21 k - - -

2.68618 4.42225 2.77519 2.73123 3.46354 2.40513 3.72494 3.29354 2.67741 2.69355 4.24690 2.90347 2.73739 3.18146 2.89801 2.37887 2.77519 2.98518 4.58477 3.61503

0.02682 4.02790 4.75025 0.61958 0.77255 0.46934 0.98194

22	2.89181 5.40262 1.74022 1.80151 4.72905 1.97581 3.76476 4.23138 2.73970 3.74004 4.57009 2.73502 3.85203 2.92333 3.31331 2.80135 3.17584 3.80742 5.90682 4.43050	22 d - - -

2.68619 4.42226 2.77521 2.73109 3.46355 2.40514 3.72496 3.29355 2.67742 2.69356 4.24691 2.90337 2.73741 3.18148 2.89802 2.37888 2.77521 2.98520 4.58478 3.61505

0.13560 2.13389 4.77543 0.35078 1.21786 0.48576 0.95510

23	2.61201 4.86282 2.68528 2.07278 4.11290 3.38761 3.62084 3.53978 2.42085 2.93669 3.93830 2.90559 2.63257 2.76593 2.89008 2.62760 2.73425 3.20299 5.35670 3.50993	24 e - - -

2.68618 4.42225 2.77519 2.73123 3.46354 2.40513 3.72494 3.29354 2.67741 2.69355 4.24690 2.90347 2.73739 3.18146 2.89801 2.37887 2.77519 2.98518 4.58477 3.61503

0.15139 4.05309 2.09462 0.61958 0.77255 0.48576 0.95510

24	2.37949 4.80695 2.81106 2.39402 4.20999 2.83479 3.64264 3.63616 1.96826 3.21173 4.02276 2.68765 3.11082 2.79173 2.82241 2.61088 2.85535 3.26725 5.41738 4.08139	25 k - - -

2.68618 4.42225 2.77519 2.73123 3.46354 2.40513 3.72494 3.29354 2.67741 2.69355 4.24690 2.90347 2.73739 3.18146 2.89801 2.37887 2.77519 2.98518 4.58477 3.61503

0.07321 3.93128 2.97644 0.61958 0.77255 0.41837 1.07328

25	2.02805 4.49286 3.04431 2.71368 4.14236 3.19894 3.89625 3.49763 2.53955 3.17828 4.04322 3.11225 1.78375 3.10888 3.07972 2.60778 2.85810 3.12170 5.45113 4.17724	26 p - - -

2.68618 4.42225 2.77519 2.73123 3.46354 2.40513 3.72494 3.29354 2.67741 2.69355 4.24690 2.90347 2.73739 3.18146 2.89801 2.37887 2.77519 2.98518 4.58477 3.61503

0.02715 4.01570 4.73805 0.61958 0.77255 0.46192 0.99446

26	2.42048 4.39695 3.18591 2.82384 4.11316 3.15834 3.96049 3.50374 2.82852 3.17894 4.03155 3.17508 3.78766 2.99089 3.18991 1.81993 1.66488 3.10317 5.44667 4.18021	27 t - - -

2.68618 4.42225 2.77519 2.73123 3.46354 2.40513 3.72494 3.29354 2.67741 2.69355 4.24690 2.90347 2.73739 3.18146 2.89801 2.37887 2.77519 2.98518 4.58477 3.61503

0.02614 4.05309 4.77543 0.61958 0.77255 0.48576 0.95510

27	2.78659 5.07008 3.04353 2.33879 4.42631 3.49635 3.60514 3.83242 1.88004 3.33461 4.14600 2.99683 2.77451 2.73163 1.88085 2.77497 2.99676 3.47502 5.46319 4.17137	28 k - - -

2.68618 4.42225 2.77519 2.73123 3.46354 2.40513 3.72494 3.29354 2.67741 2.69355 4.24690 2.90347 2.73739 3.18146 2.89801 2.37887 2.77519 2.98518 4.58477 3.61503

0.02614 4.05309 4.77543 0.61958 0.77255 0.48576 0.95510

28	2.74150 5.24603 2.20809 1.73122 4.55960 3.32193 3.64162 4.04469 2.45305 3.53404 4.31309 2.56255 3.80861 2.32801 2.74407 2.67997 2.98769 3.62354 5.68045 4.25174	29 e - - -

2.68618 4.42225 2.77519 2.73123 3.46354 2.40513 3.72494 3.29354 2.67741 2.69355 4.24690 2.90347 2.73739 3.18146 2.89801 2.37887 2.77519 2.98518 4.58477 3.61503

0.02614 4.05309 4.77543 0.61958 0.77255 0.48576 0.95510

29	2.80576 5.20871 2.58171 1.59913 4.48771 3.36966 3.15549 3.97488 2.39319 3.47957 4.29197 2.84664 3.85595 2.14029 2.81700 2.75654 3.04567 3.59169 5.61510 4.22659	30 e - - -

2.68618 4.42225 2.77519 2.73123 3.46354 2.40513 3.72494 3.29354 2.67741 2.69355 4.24690 2.90347 2.73739 3.18146 2.89801 2.37887 2.77519 2.98518 4.58477 3.61503

0.02614 4.05309 4.77543 0.61958 0.77255 0.48576 0.95510

30	3.15347 4.54442 4.61194 4.07315 3.19235 4.33620 4.69894 1.76330 3.89945 1.63679 1.54169 4.33674 4.61366 4.12434 4.07244 3.68845 3.39614 2.13662 5.17296 4.03677	31 m - - -

2.68618 4.42225 2.77519 2.73123 3.46354 2.40513 3.72494 3.29354 2.67741 2.69355 4.24690 2.90347 2.73739 3.18146 2.89801 2.37887 2.77519 2.98518 4.58477 3.61503

0.02614 4.05309 4.77543 0.61958 0.77255 0.48576 0.95510

31	3.13445 4.44504 4.85391 4.36483 3.57688 4.48392 5.07395 1.10729 4.24798 2.06244 3.37982 4.57817 4.79519 4.50999 4.43993 3.87935 3.40259 1.34356 5.56522 4.36311	32 i - - -

2.68618 4.42225 2.77519 2.73123 3.46354 2.40513 3.72494 3.29354 2.67741 2.69355 4.24690 2.90347 2.73739 3.18146 2.89801 2.37887 2.77519 2.98518 4.58477 3.61503

0.02614 4.05309 4.77543 0.61958 0.77255 0.48576 0.95510

32	2.19711 5.13500 1.81324 2.24466 4.47458 3.29819 3.68728 3.94373 2.55289 3.47763 4.27860 2.56950 3.81431 2.58170 3.06919 2.51271 2.99537 3.54351 5.65981 4.24415	33 d - - -

2.68618 4.42225 2.77519 2.73123 3.46354 2.40513 3.72494 3.29354 2.67741 2.69355 4.24690 2.90347 2.73739 3.18146 2.89801 2.37887 2.77519 2.98518 4.58477 3.61503

0.02614 4.05309 4.77543 0.61958 0.77255 0.48576 0.95510

33	1.95465 2.86548 3.68254 3.16345 3.62135 3.27748 4.02358 2.66848 3.09736 2.68965 3.58471 3.40631 3.85126 3.38245 3.40202 2.05939 2.38177 2.62349 5.07300 3.86134	34 a - - -

2.68618 4.42225 2.77519 2.73123 3.46354 2.40513 3.72494 3.29354 2.67741 2.69355 4.24690 2.90347 2.73739 3.18146 2.89801 2.37887 2.77519 2.98518 4.58477 3.61503

0.02614 4.05309 4.77543 0.61958 0.77255 0.48576 0.95510

34	3.67762 4.91664 4.62646 4.31786 1.49683 4.41042 3.48828 3.37482 4.16562 2.70249 3.97293 4.08441 4.72246 4.14184 4.20665 3.78272 3.90086 3.29876 3.62912 0.96979	35 y - - -

2.68618 4.42225 2.77519 2.73123 3.46354 2.40513 3.72494 3.29354 2.67741 2.69355 4.24690 2.90347 2.73739 3.18146 2.89801 2.37887 2.77519 2.98518 4.58477 3.61503

0.02614 4.05309 4.77543 0.61958 0.77255 0.48576 0.95510

35	2.73355 4.38155 3.64414 2.32391 3.51907 3.75896 4.04418 1.97261 3.03380 2.20115 3.42188 3.50677 4.12799 3.35989 3.37724 3.02755 2.97095 1.86105 5.09080 3.85752	36 v - - -

2.68618 4.42225 2.77519 2.73123 3.46354 2.40513 3.72494 3.29354 2.67741 2.69355 4.24690 2.90347 2.73739 3.18146 2.89801 2.37887 2.77519 2.98518 4.58477 3.61503

0.02614 4.05309 4.77543 0.61958 0.77255 0.48576 0.95510

36	2.76306 5.14592 2.93012 2.11204 4.51080 3.45853 3.59005 3.94150 1.89037 3.41409 4.20333 2.67207 3.86071 2.28537 2.18379 2.73038 2.97699 3.55223 5.52338 4.19705	37 k - - -

2.68618 4.42225 2.77519 2.73123 3.46354 2.40513 3.72494 3.29354 2.67741 2.69355 4.24690 2.90347 2.73739 3.18146 2.89801 2.37887 2.77519 2.98518 4.58477 3.61503

0.02614 4.05309 4.77543 0.61958 0.77255 0.48576 0.95510

37	2.65415 4.33801 3.81102 3.35183 3.63354 3.57199 4.24690 2.00760 3.23159 2.43818 3.55797 3.63314 4.10561 3.58650 3.51771 2.95110 1.52496 2.22979 5.25375 4.01651	38 t - - -

2.68618 4.42225 2.77519 2.73123 3.46354 2.40513 3.72494 3.29354 2.67741 2.69355 4.24690 2.90347 2.73739 3.18146 2.89801 2.37887 2.77519 2.98518 4.58477 3.61503

0.02614 4.05309 4.77543 0.61958 0.77255 0.48576 0.95510

38	2.82329 2.39575 4.36616 3.85592 3.28563 3.84283 4.42191 2.28911 3.66306 1.26287 3.24983 4.01316 4.30066 3.93482 3.83427 3.23585 3.12536 2.19414 5.03375 3.81122	39 l - - -

2.68618 4.42225 2.77519 2.73123 3.46354 2.40513 3.72494 3.29354 2.67741 2.69355 4.24690 2.90347 2.73739 3.18146 2.89801 2.37887 2.77519 2.98518 4.58477 3.61503

0.02614 4.05309 4.77543 0.61958 0.77255 0.48576 0.95510

39	1.02437 4.15745 3.56990 3.29064 4.26676 2.97663 4.30607 3.53079 3.30570 3.33828 4.20433 3.39005 3.73383 3.60365 3.58442 2.20704 2.52733 3.04912 5.65019 4.45014	40 a - - -

2.68618 4.42225 2.77519 2.73123 3.46354 2.40513 3.72494 3.29354 2.67741 2.69355 4.24690 2.90347 2.73739 3.18146 2.89801 2.37887 2.77519 2.98518 4.58477 3.61503

0.02614 4.05309 4.77543 0.61958 0.77255 0.48576 0.95510

40	2.64171 4.89702 2.97101 2.36634 4.17731 3.42699 3.60446 3.59553 1.99168 3.16301 3.47706 2.94224 3.82168 2.20537 2.71446 2.50282 2.65720 3.25379 5.36539 4.03464	41 k - - -

2.68618 4.42225 2.77519 2.73123 3.46354 2.40513 3.72494 3.29354 2.67741 2.69355 4.24690 2.90347 2.73739 3.18146 2.89801 2.37887 2.77519 2.98518 4.58477 3.61503

0.02614 4.05309 4.77543 0.61958 0.77255 0.48576 0.95510

41	2.35946 4.23185 3.74007 3.22761 3.55598 2.39950 4.08984 2.22987 3.16148 2.52007 3.49792 3.52219 4.01138 3.45939 3.46382 2.84934 2.86776 1.72352 5.07700 3.85682	42 v - - -

2.68618 4.42225 2.77519 2.73123 3.46354 2.40513 3.72494 3.29354 2.67741 2.69355 4.24690 2.90347 2.73739 3.18146 2.89801 2.37887 2.77519 2.98518 4.58477 3.61503

0.02614 4.05309 4.77543 0.61958 0.77255 0.48576 0.95510

42	3.17790 4.52801 4.74003 4.20288 3.27463 4.43683 4.83757 1.92374 4.05957 1.13904 3.09119 4.45624 4.69926 4.26516 4.23199 3.79413 3.42295 1.55157 5.28049 4.13841	43 l - - -

2.68618 4.42225 2.77519 2.73123 3.46354 2.40513 3.72494 3.29354 2.67741 2.69355 4.24690 2.90347 2.73739 3.18146 2.89801 2.37887 2.77519 2.98518 4.58477 3.61503

0.02614 4.05309 4.77543 0.61958 0.77255 0.48576 0.95510

43	2.24687 4.50176 2.86894 2.68531 4.41774 1.46670 4.01798 3.86700 2.94609 3.50574 4.33664 2.29813 3.77562 3.23308 3.34974 2.56394 2.88355 3.36669 5.70471 4.40259	44 g - - -

2.68618 4.42225 2.77519 2.73123 3.46354 2.40513 3.72494 3.29354 2.67741 2.69355 4.24690 2.90347 2.73739 3.18146 2.89801 2.37887 2.77519 2.98518 4.58477 3.61503

0.02614 4.05309 4.77543 0.61958 0.77255 0.48576 0.95510

44	2.59167 4.29394 3.56761 3.02714 3.33622 3.53814 3.87019 2.25932 2.92456 2.48550 3.43827 3.38122 3.96880 3.25498 3.23952 1.80598 2.85532 2.55046 4.13737 3.53214	45 s - - -

2.68620 4.42227 2.77522 2.73126 3.46356 2.40515 3.72497 3.29356 2.67743 2.69357 4.24692 2.90349 2.73742 3.18149 2.89803 2.37861 2.77522 2.98521 4.58479 3.61506

0.21103 1.70475 4.77543 0.27625 1.42138 0.48576 0.95510

45	2.61621 4.50186 3.25437 2.04318 3.63816 3.52913 3.75758 2.51695 2.64776 2.47972 3.16271 3.16390 3.91088 2.87197 3.04668 2.76204 2.84748 2.59179 5.06637 3.80242	47 e - - -

2.68618 4.42225 2.77519 2.73123 3.46354 2.40513 3.72494 3.29354 2.67741 2.69355 4.24690 2.90347 2.73739 3.18146 2.89801 2.37887 2.77519 2.98518 4.58477 3.61503

0.02614 4.05309 4.77543 0.61958 0.77255 0.48576 0.95510

46	2.98127 5.41420 2.14392 1.03573 4.72590 3.28532 3.82842 4.21821 2.80014 3.76670 4.64191 2.78847 3.89944 3.00460 3.33668 2.89210 3.27252 3.82469 5.90407 4.46365	48 e - - -

2.68618 4.42225 2.77519 2.73123 3.46354 2.40513 3.72494 3.29354 2.67741 2.69355 4.24690 2.90347 2.73739 3.18146 2.89801 2.37887 2.77519 2.98518 4.58477 3.61503

0.02614 4.05309 4.77543 0.61958 0.77255 0.48576 0.95510

47	3.11886 5.25606 2.57879 0.79330 4.62666 3.39863 4.00594 4.11708 2.91011 3.70676 4.67670 3.05037 4.01297 3.22029 3.33489 3.08511 3.42573 3.78371 5.76410 4.49110	49 e - - -

2.68618 4.42225 2.77519 2.73123 3.46354 2.40513 3.72494 3.29354 2.67741 2.69355 4.24690 2.90347 2.73739 3.18146 2.89801 2.37887 2.77519 2.98518 4.58477 3.61503

0.02614 4.05309 4.77543 0.61958 0.77255 0.48576 0.95510

48	0.78178 4.29150 3.65453 3.45845 4.24053 3.11039 4.45795 3.39370 3.47958 3.26662 4.28157 3.55966 3.86708 3.80479 3.71511 2.64176 2.93185 3.01654 5.64947 4.47108	50 a - - -

2.68618 4.42225 2.77519 2.73123 3.46354 2.40513 3.72494 3.29354 2.67741 2.69355 4.24690 2.90347 2.73739 3.18146 2.89801 2.37887 2.77519 2.98518 4.58477 3.61503

0.02614 4.05309 4.77543 0.61958 0.77255 0.48576 0.95510

49	3.08656 5.19393 3.47107 2.82591 4.65219 3.68706 3.64585 4.00500 1.11396 3.44556 4.32102 3.22713 4.06386 2.78242 1.83037 3.08162 3.25587 3.68644 5.48184 4.31547	51 k - - -

2.68618 4.42225 2.77519 2.73123 3.46354 2.40513 3.72494 3.29354 2.67741 2.69355 4.24690 2.90347 2.73739 3.18146 2.89801 2.37887 2.77519 2.98518 4.58477 3.61503

0.02614 4.05309 4.77543 0.61958 0.77255 0.48576 0.95510

50	2.77832 5.00519 3.15695 2.55306 4.34035 3.52405 3.60314 3.73488 1.51578 3.25794 3.67546 2.92128 3.90062 2.60824 2.20619 2.78324 2.81543 3.39672 5.40383 4.12819	52 k - - -

2.68618 4.42225 2.77519 2.73123 3.46354 2.40513 3.72494 3.29354 2.67741 2.69355 4.24690 2.90347 2.73739 3.18146 2.89801 2.37887 2.77519 2.98518 4.58477 3.61503

0.02614 4.05309 4.77543 0.61958 0.77255 0.48576 0.95510

51	2.81149 5.07741 3.08874 2.53816 4.46248 3.51010 3.61127 3.86541 1.59117 3.35786 4.17344 2.71246 3.90752 2.73843 2.01480 2.48556 3.02166 3.50617 5.47183 4.19400	53 k - - -

2.68618 4.42225 2.77519 2.73123 3.46354 2.40513 3.72494 3.29354 2.67741 2.69355 4.24690 2.90347 2.73739 3.18146 2.89801 2.37887 2.77519 2.98518 4.58477 3.61503

0.02614 4.05309 4.77543 0.61958 0.77255 0.48576 0.95510

52	3.16139 4.50551 4.74285 4.20837 3.28427 4.42281 4.82316 1.20482 4.04933 1.73384 2.30330 4.44994 4.68862 4.26535 4.21251 3.78304 3.40526 1.93214 5.27044 4.12835	54 i - - -

2.68618 4.42225 2.77519 2.73123 3.46354 2.40513 3.72494 3.29354 2.67741 2.69355 4.24690 2.90347 2.73739 3.18146 2.89801 2.37887 2.77519 2.98518 4.58477 3.61503

0.02614 4.05309 4.77543 0.61958 0.77255 0.48576 0.95510

53	3.51777 4.93319 4.10954 3.87597 2.32254 4.04419 3.71764 3.50291 3.72851 2.90932 4.15976 3.95265 4.51531 3.99054 3.87373 3.63513 3.80616 3.38243 3.97211 0.71120	55 y - - -

2.68618 4.42225 2.77519 2.73123 3.46354 2.40513 3.72494 3.29354 2.67741 2.69355 4.24690 2.90347 2.73739 3.18146 2.89801 2.37887 2.77519 2.98518 4.58477 3.61503

0.02614 4.05309 4.77543 0.61958 0.77255 0.48576 0.95510

54	1.63011 4.41720 3.08327 2.76302 4.26373 3.10369 3.97551 3.68479 2.87301 3.33537 4.15906 2.36305 3.75727 3.18553 3.27041 1.84736 2.80385 3.22173 5.56705 4.28509	56 a - - -

2.68618 4.42225 2.77519 2.73123 3.46354 2.40513 3.72494 3.29354 2.67741 2.69355 4.24690 2.90347 2.73739 3.18146 2.89801 2.37887 2.77519 2.98518 4.58477 3.61503

0.02614 4.05309 4.77543 0.61958 0.77255 0.48576 0.95510

55	2.79945 2.77485 4.46928 3.90672 2.88783 3.99700 4.40336 1.92394 3.78041 2.13327 3.24867 4.06607 4.35518 3.98667 3.93173 3.32363 3.04946 1.38069 4.97962 3.77355	57 v - - -

2.68618 4.42225 2.77519 2.73123 3.46354 2.40513 3.72494 3.29354 2.67741 2.69355 4.24690 2.90347 2.73739 3.18146 2.89801 2.37887 2.77519 2.98518 4.58477 3.61503

0.02614 4.05309 4.77543 0.61958 0.77255 0.48576 0.95510

56	2.23688 2.91486 3.81205 3.44704 4.10861 2.99289 4.32270 3.46222 3.36128 3.24052 4.11697 3.47014 3.74368 3.66099 3.61331 1.06805 2.72714 3.00195 5.51984 4.30514	58 s - - -

2.68618 4.42225 2.77519 2.73123 3.46354 2.40513 3.72494 3.29354 2.67741 2.69355 4.24690 2.90347 2.73739 3.18146 2.89801 2.37887 2.77519 2.98518 4.58477 3.61503

0.02614 4.05309 4.77543 0.61958 0.77255 0.48576 0.95510

57	2.32864 2.58890 3.94507 3.53321 3.89368 3.13267 4.32755 2.97851 3.40253 2.90673 3.86439 3.56933 3.83887 3.70660 3.63315 2.57341 1.29032 2.66094 5.37497 4.18038	59 t - - -

2.68618 4.42225 2.77519 2.73123 3.46354 2.40513 3.72494 3.29354 2.67741 2.69355 4.24690 2.90347 2.73739 3.18146 2.89801 2.37887 2.77519 2.98518 4.58477 3.61503

0.02614 4.05309 4.77543 0.61958 0.77255 0.48576 0.95510

58	2.67384 4.92277 2.91839 2.13600 4.22257 3.42068 3.62443 3.63847 2.18699 3.20353 4.01523 2.94380 3.83607 2.76472 2.45010 2.58355 2.09776 3.29479 5.40065 4.07340	60 t - - -

2.68618 4.42225 2.77519 2.73123 3.46354 2.40513 3.72494 3.29354 2.67741 2.69355 4.24690 2.90347 2.73739 3.18146 2.89801 2.37887 2.77519 2.98518 4.58477 3.61503

0.02614 4.05309 4.77543 0.61958 0.77255 0.48576 0.95510

59	2.56120 3.45647 3.43453 2.86744 3.45412 3.52497 3.79644 2.63783 2.79584 2.52634 3.41862 3.27149 3.91633 3.11902 2.65612 2.63337 2.22517 2.58002 4.89309 3.27240	61 t - - -

2.68618 4.42225 2.77519 2.73123 3.46354 2.40513 3.72494 3.29354 2.67741 2.69355 4.24690 2.90347 2.73739 3.18146 2.89801 2.37887 2.77519 2.98518 4.58477 3.61503

0.02614 4.05309 4.77543 0.61958 0.77255 0.48576 0.95510

60	3.59990 4.90864 4.46271 4.14088 1.84672 4.32322 3.51823 3.34926 3.99492 2.70439 3.97037 4.01981 4.66419 4.05703 4.09124 3.70779 3.83570 3.26532 3.68640 0.85068	62 y - - -

2.68618 4.42225 2.77519 2.73123 3.46354 2.40513 3.72494 3.29354 2.67741 2.69355 4.24690 2.90347 2.73739 3.18146 2.89801 2.37887 2.77519 2.98518 4.58477 3.61503

0.02614 4.05309 4.77543 0.61958 0.77255 0.48576 0.95510

61	2.62191 4.39355 3.39273 2.83902 2.70993 3.56206 3.75281 2.88843 2.77653 2.49412 3.47824 3.25712 3.94427 2.39585 3.13873 2.80926 2.47236 2.66774 4.79228 2.83836	63 q - - -

2.68618 4.42225 2.77519 2.73123 3.46354 2.40513 3.72494 3.29354 2.67741 2.69355 4.24690 2.90347 2.73739 3.18146 2.89801 2.37887 2.77519 2.98518 4.58477 3.61503

0.02614 4.05309 4.77543 0.61958 0.77255 0.48576 0.95510

62	1.72279 4.23092 3.38696 3.19701 4.42226 1.21255 4.32610 3.79450 3.33811 3.53062 4.38324 3.35444 3.74626 3.61403 3.63450 2.47356 2.79820 3.24361 5.75203 4.55777	64 g - - -

2.68618 4.42225 2.77519 2.73123 3.46354 2.40513 3.72494 3.29354 2.67741 2.69355 4.24690 2.90347 2.73739 3.18146 2.89801 2.37887 2.77519 2.98518 4.58477 3.61503

0.02614 4.05309 4.77543 0.61958 0.77255 0.48576 0.95510

63	3.49677 4.84667 4.48678 4.23877 0.75674 4.13762 3.96991 3.00189 4.14213 2.33177 3.70732 4.22709 4.59457 4.26106 4.21706 3.75519 3.79923 3.00531 4.17142 2.51824	65 f - - -

2.68618 4.42225 2.77519 2.73123 3.46354 2.40513 3.72494 3.29354 2.67741 2.69355 4.24690 2.90347 2.73739 3.18146 2.89801 2.37887 2.77519 2.98518 4.58477 3.61503

0.02614 4.05309 4.77543 0.61958 0.77255 0.48576 0.95510

64	2.71121 4.83396 2.78855 2.54443 4.36210 1.76193 3.84571 3.85555 2.61497 3.42932 4.28826 3.02349 3.87267 1.84044 2.99024 2.75515 3.03942 3.46077 5.57532 4.25872	66 g - - -

2.68618 4.42225 2.77519 2.73123 3.46354 2.40513 3.72494 3.29354 2.67741 2.69355 4.24690 2.90347 2.73739 3.18146 2.89801 2.37887 2.77519 2.98518 4.58477 3.61503

0.02614 4.05309 4.77543 0.61958 0.77255 0.48576 0.95510

65	1.67782 2.16784 4.08226 3.61859 3.68593 3.28571 4.32848 2.59512 3.50776 2.64891 3.63611 3.66736 3.93396 3.76461 3.72305 2.69810 2.81021 2.00470 5.22538 4.03446	67 a - - -

2.68618 4.42225 2.77519 2.73123 3.46354 2.40513 3.72494 3.29354 2.67741 2.69355 4.24690 2.90347 2.73739 3.18146 2.89801 2.37887 2.77519 2.98518 4.58477 3.61503

0.02614 4.05309 4.77543 0.61958 0.77255 0.48576 0.95510

66	2.59297 4.55275 3.15898 2.30221 3.74467 3.47114 3.69621 2.96293 2.54173 2.25282 3.64847 3.08471 3.86317 2.91159 2.74254 2.55355 2.50526 2.84456 5.10214 3.82399	68 l - - -

2.68618 4.42225 2.77519 2.73123 3.46354 2.40513 3.72494 3.29354 2.67741 2.69355 4.24690 2.90347 2.73739 3.18146 2.89801 2.37887 2.77519 2.98518 4.58477 3.61503

0.02614 4.05309 4.77543 0.61958 0.77255 0.48576 0.95510

67	3.10169 4.42775 4.77489 4.22651 3.35337 4.41146 4.82900 1.34857 4.10870 1.83973 2.35668 4.44845 4.67689 4.29988 4.26929 3.75867 3.34292 1.57318 5.29287 4.15525	69 i - - -

2.68618 4.42225 2.77519 2.73123 3.46354 2.40513 3.72494 3.29354 2.67741 2.69355 4.24690 2.90347 2.73739 3.18146 2.89801 2.37887 2.77519 2.98518 4.58477 3.61503

0.02614 4.05309 4.77543 0.61958 0.77255 0.48576 0.95510

68	2.62738 4.92926 2.25369 2.33913 4.25508 3.33781 3.22937 3.70038 2.46076 3.26594 4.06476 2.87352 3.54940 2.78695 2.94382 2.25372 2.27054 3.32969 5.47054 4.09922	70 d - - -

2.68618 4.42225 2.77519 2.73123 3.46354 2.40513 3.72494 3.29354 2.67741 2.69355 4.24690 2.90347 2.73739 3.18146 2.89801 2.37887 2.77519 2.98518 4.58477 3.61503

0.02614 4.05309 4.77543 0.61958 0.77255 0.48576 0.95510

69	2.73258 4.97183 2.74302 1.48816 4.14219 3.39953 3.22069 3.24932 2.44216 3.21684 4.06647 2.91934 3.85914 2.83533 2.86502 2.73465 2.97423 3.30904 5.40240 4.02522	71 e - - -

2.68618 4.42225 2.77519 2.73123 3.46354 2.40513 3.72494 3.29354 2.67741 2.69355 4.24690 2.90347 2.73739 3.18146 2.89801 2.37887 2.77519 2.98518 4.58477 3.61503

0.02614 4.05309 4.77543 0.61958 0.77255 0.48576 0.95510

70	2.63898 5.17283 2.43904 1.71704 4.48158 3.03735 3.64459 3.95430 2.46623 3.46740 4.25303 2.79916 3.29027 2.26305 2.97123 2.67036 2.96647 3.54961 5.63312 4.21671	72 e - - -

2.68618 4.42225 2.77519 2.73123 3.46354 2.40513 3.72494 3.29354 2.67741 2.69355 4.24690 2.90347 2.73739 3.18146 2.89801 2.37887 2.77519 2.98518 4.58477 3.61503

0.02614 4.05309 4.77543 0.61958 0.77255 0.48576 0.95510

71	2.62115 4.55777 3.16676 2.14408 3.76194 3.48836 3.76203 2.96862 2.61871 2.32394 3.65883 3.12830 3.90042 2.97966 3.02132 2.74749 2.29099 2.55998 5.15900 3.87992	73 e - - -

2.68618 4.42225 2.77519 2.73123 3.46354 2.40513 3.72494 3.29354 2.67741 2.69355 4.24690 2.90347 2.73739 3.18146 2.89801 2.37887 2.77519 2.98518 4.58477 3.61503

0.02614 4.05309 4.77543 0.61958 0.77255 0.48576 0.95510

72	2.08891 4.21229 3.52562 3.12358 4.12510 3.05684 4.14508 3.40412 3.10015 3.18154 4.04341 3.32683 3.75220 3.41243 3.42870 1.30993 2.41962 2.65820 5.50213 4.27977	74 s - - -

2.68618 4.42225 2.77519 2.73123 3.46354 2.40513 3.72494 3.29354 2.67741 2.69355 4.24690 2.90347 2.73739 3.18146 2.89801 2.37887 2.77519 2.98518 4.58477 3.61503

0.02614 4.05309 4.77543 0.61958 0.77255 0.48576 0.95510

73	2.59954 4.22471 2.96065 1.96347 3.97915 3.41408 3.41142 3.41331 2.43365 3.02411 3.84721 2.61372 3.81469 2.79186 2.88332 2.63999 2.76055 3.09899 5.26149 3.07365	75 e - - -

2.68618 4.42225 2.77519 2.73123 3.46354 2.40513 3.72494 3.29354 2.67741 2.69355 4.24690 2.90347 2.73739 3.18146 2.89801 2.37887 2.77519 2.98518 4.58477 3.61503

0.02614 4.05309 4.77543 0.61958 0.77255 0.48576 0.95510

74	3.14556 5.10845 3.33302 2.92530 4.53640 3.61137 3.87180 3.97797 0.81520 3.50545 4.46065 3.34046 4.10183 3.05555 2.48253 3.17778 3.38712 3.67684 5.51940 4.35987	76 k - - -

2.68618 4.42225 2.77519 2.73123 3.46354 2.40513 3.72494 3.29354 2.67741 2.69355 4.24690 2.90347 2.73739 3.18146 2.89801 2.37887 2.77519 2.98518 4.58477 3.61503

0.02614 4.05309 4.77543 0.61958 0.77255 0.48576 0.95510

75	3.12912 4.47053 4.76192 4.19674 1.95473 4.35996 4.60477 1.86309 4.07102 1.36159 2.97334 4.38552 4.60223 4.18265 4.18196 3.69100 3.35512 2.01013 4.96714 3.75565	77 l - - -

2.68618 4.42225 2.77519 2.73123 3.46354 2.40513 3.72494 3.29354 2.67741 2.69355 4.24690 2.90347 2.73739 3.18146 2.89801 2.37887 2.77519 2.98518 4.58477 3.61503

0.02614 4.05309 4.77543 0.61958 0.77255 0.48576 0.95510

76	2.91792 5.18854 3.14343 2.16799 4.59918 3.56999 3.60831 3.98069 1.54566 3.43371 4.26005 3.05985 3.95656 2.73219 1.81260 2.89209 3.10777 3.62492 5.50859 4.26216	78 k - - -

2.68618 4.42225 2.77519 2.73123 3.46354 2.40513 3.72494 3.29354 2.67741 2.69355 4.24690 2.90347 2.73739 3.18146 2.89801 2.37887 2.77519 2.98518 4.58477 3.61503

0.02614 4.05309 4.77543 0.61958 0.77255 0.48576 0.95510

77	2.51570 5.15805 2.01605 2.01360 4.51578 2.18230 3.70782 3.98751 2.59995 3.52091 4.32632 2.79087 3.81834 2.85372 3.12907 2.47162 3.02174 3.58091 5.70517 4.28084	79 e - - -

2.68621 4.42132 2.77523 2.73126 3.46342 2.40516 3.72498 3.29357 2.67744 2.69351 4.24693 2.90350 2.73743 3.18149 2.89783 2.37890 2.77523 2.98521 4.58480 3.61506

0.10288 2.41537 4.77543 0.81631 0.58350 0.48576 0.95510

78	3.18360 4.57920 4.64376 4.14234 3.22255 4.34513 4.77943 2.09545 3.96416 0.99233 3.06856 4.39291 4.65414 4.20211 4.13679 3.72917 3.44509 1.76536 5.23289 4.06472	84 l - - -

2.68618 4.42225 2.77519 2.73123 3.46354 2.40513 3.72494 3.29354 2.67741 2.69355 4.24690 2.90347 2.73739 3.18146 2.89801 2.37887 2.77519 2.98518 4.58477 3.61503

0.02614 4.05309 4.77543 0.61958 0.77255 0.48576 0.95510

79	2.60625 4.65095 2.92795 2.64801 4.24697 3.25690 3.86917 3.66815 2.37861 3.30044 4.16231 3.08008 1.53354 3.07073 2.96628 2.52420 2.95647 3.28475 5.50875 4.21503	85 p - - -

2.68618 4.42225 2.77519 2.73123 3.46354 2.40513 3.72494 3.29354 2.67741 2.69355 4.24690 2.90347 2.73739 3.18146 2.89801 2.37887 2.77519 2.98518 4.58477 3.61503

0.02614 4.05309 4.77543 0.61958 0.77255 0.48576 0.95510

80	2.70874 5.00882 2.34883 2.31502 4.41426 1.80848 3.72776 3.87588 2.58085 3.43354 4.24979 2.47391 3.82418 2.88344 2.79509 2.69751 2.99325 3.48244 5.62388 4.23819	86 g - - -

2.68618 4.42225 2.77519 2.73123 3.46354 2.40513 3.72494 3.29354 2.67741 2.69355 4.24690 2.90347 2.73739 3.18146 2.89801 2.37887 2.77519 2.98518 4.58477 3.61503

0.02614 4.05309 4.77543 0.61958 0.77255 0.48576 0.95510

81	2.45624 4.32273 4.22384 3.85113 3.74858 3.72367 4.67834 2.06422 3.74879 2.42839 3.62666 3.99916 4.30547 4.07387 3.97447 3.16409 3.11969 0.99176 5.51229 4.28067	87 v - - -

2.68618 4.42225 2.77519 2.73123 3.46354 2.40513 3.72494 3.29354 2.67741 2.69355 4.24690 2.90347 2.73739 3.18146 2.89801 2.37887 2.77519 2.98518 4.58477 3.61503

0.02614 4.05309 4.77543 0.61958 0.77255 0.48576 0.95510

82	2.64617 4.46069 3.38383 2.80116 3.61653 3.55769 3.75050 2.95716 2.39261 2.05354 3.54496 3.22713 3.93625 2.81899 2.57034 2.81497 2.87085 2.28510 5.00732 3.76811	88 l - - -

2.68618 4.42225 2.77519 2.73123 3.46354 2.40513 3.72494 3.29354 2.67741 2.69355 4.24690 2.90347 2.73739 3.18146 2.89801 2.37887 2.77519 2.98518 4.58477 3.61503

0.02614 4.05309 4.77543 0.61958 0.77255 0.48576 0.95510

83	3.63280 4.84392 4.74368 4.41881 1.23328 4.41856 3.47642 3.34913 4.23900 2.69883 3.92642 4.10824 4.71431 4.17020 4.23458 3.77351 3.84698 3.25550 1.82933 1.74097	89 f - - -

2.68618 4.42225 2.77519 2.73123 3.46354 2.40513 3.72494 3.29354 2.67741 2.69355 4.24690 2.90347 2.73739 3.18146 2.89801 2.37887 2.77519 2.98518 4.58477 3.61503

0.02614 4.05309 4.77543 0.61958 0.77255 0.48576 0.95510

84	3.09091 4.42956 4.75854 4.27520 3.60328 4.39469 5.00657 1.56134 4.15433 2.12463 3.42207 4.49172 4.74006 4.43791 4.36109 3.79041 3.36739 0.97386 5.55585 4.33425	90 v - - -

2.68618 4.42225 2.77519 2.73123 3.46354 2.40513 3.72494 3.29354 2.67741 2.69355 4.24690 2.90347 2.73739 3.18146 2.89801 2.37887 2.77519 2.98518 4.58477 3.61503

0.02614 4.05309 4.77543 0.61958 0.77255 0.48576 0.95510

85	2.84167 4.38318 4.06107 3.54959 3.26571 3.87082 4.26404 2.19161 3.37331 1.20688 3.22900 3.83168 4.27312 3.69865 3.60716 2.84919 3.10966 2.25107 4.99965 3.75624	91 l - - -

2.68618 4.42225 2.77519 2.73123 3.46354 2.40513 3.72494 3.29354 2.67741 2.69355 4.24690 2.90347 2.73739 3.18146 2.89801 2.37887 2.77519 2.98518 4.58477 3.61503

0.02614 4.05309 4.77543 0.61958 0.77255 0.48576 0.95510

86	3.00861 4.73204 3.63590 3.50360 4.47744 3.40950 4.56328 4.07302 3.57709 3.67874 4.73862 3.79046 0.55117 3.96030 3.79917 3.18252 3.46506 3.69715 5.61090 4.60168	92 P - - -

2.68618 4.42225 2.77519 2.73123 3.46354 2.40513 3.72494 3.29354 2.67741 2.69355 4.24690 2.90347 2.73739 3.18146 2.89801 2.37887 2.77519 2.98518 4.58477 3.61503

0.02614 4.05309 4.77543 0.61958 0.77255 0.48576 0.95510

87	3.19336 5.33798 0.68988 2.46558 4.74155 3.35626 4.10344 4.37292 3.22378 3.95042 4.93599 3.02970 4.02214 3.34535 3.76185 3.13775 3.53700 3.99400 5.87405 4.58916	93 D - - -

2.68618 4.42225 2.77519 2.73123 3.46354 2.40513 3.72494 3.29354 2.67741 2.69355 4.24690 2.90347 2.73739 3.18146 2.89801 2.37887 2.77519 2.98518 4.58477 3.61503

0.02614 4.05309 4.77543 0.61958 0.77255 0.48576 0.95510

88	2.41286 4.33372 3.37326 3.20115 4.26125 3.06073 4.30412 3.81015 3.28725 3.51765 4.43088 3.39419 3.81522 3.62296 3.56509 0.84795 2.90719 3.30519 5.61989 4.34677	94 s - - -

2.68618 4.42225 2.77519 2.73123 3.46354 2.40513 3.72494 3.29354 2.67741 2.69355 4.24690 2.90347 2.73739 3.18146 2.89801 2.37887 2.77519 2.98518 4.58477 3.61503

0.11163 4.05309 2.42754 0.61958 0.77255 0.48576 0.95510

89	3.48838 4.82077 4.37204 4.02643 1.78478 4.24150 3.48028 3.24428 3.88755 2.61594 3.86838 3.94299 4.58152 3.96619 4.00086 3.61395 3.72278 3.15734 3.66124 0.97234	95 y - - -

2.68618 4.42225 2.77519 2.73123 3.46354 2.40513 3.72494 3.29354 2.67741 2.69355 4.24690 2.90347 2.73739 3.18146 2.89801 2.37887 2.77519 2.98518 4.58477 3.61503

0.02844 3.96990 4.69225 0.61958 0.77255 0.56947 0.83430

90	3.12052 4.43666 4.76914 4.23358 3.36402 4.45579 4.89102 1.40129 4.11708 1.54263 3.16762 4.47837 4.71178 4.32909 4.29739 3.81369 3.36520 1.49031 5.35103 4.19722	96 i - - -

2.68618 4.42225 2.77519 2.73123 3.46354 2.40513 3.72494 3.29354 2.67741 2.69355 4.24690 2.90347 2.73739 3.18146 2.89801 2.37887 2.77519 2.98518 4.58477 3.61503

0.02844 3.96990 4.69225 0.61958 0.77255 0.56947 0.83430

91	2.74866 4.93214 1.34214 2.35756 4.24293 3.26622 3.82413 3.53460 2.77790 3.29068 4.23536 2.88957 3.85415 3.01895 3.27294 2.76377 3.06379 2.76222 5.58970 4.18305	97 d - - -

2.68618 4.42225 2.77519 2.73123 3.46354 2.40513 3.72494 3.29354 2.67741 2.69355 4.24690 2.90347 2.73739 3.18146 2.89801 2.37887 2.77519 2.98518 4.58477 3.61503

0.02844 3.96990 4.69225 0.61958 0.77255 0.43676 1.03881

92	2.59828 4.66196 2.78222 2.51464 3.89941 3.41724 3.68117 3.28548 2.51035 2.93385 3.78029 3.01084 2.69808 2.77989 2.74000 2.67230 2.84175 2.25904 5.21854 3.91438	98 v - - -

2.68618 4.42225 2.77519 2.73123 3.46354 2.40513 3.72494 3.29354 2.67741 2.69355 4.24690 2.90347 2.73739 3.18146 2.89801 2.37887 2.77519 2.98518 4.58477 3.61503

0.02614 4.05309 4.77543 0.61958 0.77255 0.48576 0.95510

93	2.62145 4.95542 2.87405 2.04317 4.24633 2.91207 3.59217 3.68527 2.11530 3.23308 4.02071 2.88987 3.39619 2.61911 2.68147 2.61621 2.85242 2.95595 5.42117 4.06054	99 e - - -

2.68618 4.42225 2.77519 2.73123 3.46354 2.40513 3.72494 3.29354 2.67741 2.69355 4.24690 2.90347 2.73739 3.18146 2.89801 2.37887 2.77519 2.98518 4.58477 3.61503

0.02614 4.05309 4.77543 0.61958 0.77255 0.48576 0.95510

94	2.63816 4.89388 2.62702 2.37193 3.80845 3.38788 3.62713 3.57613 2.31051 3.15917 3.97302 1.94886 3.81276 2.78095 2.90174 2.64820 2.87507 3.23829 5.34240 3.44965	100 n - - -

2.68618 4.42225 2.77519 2.73123 3.46354 2.40513 3.72494 3.29354 2.67741 2.69355 4.24690 2.90347 2.73739 3.18146 2.89801 2.37887 2.77519 2.98518 4.58477 3.61503

0.02614 4.05309 4.77543 0.61958 0.77255 0.48576 0.95510

95	2.94536 5.11497 2.99968 2.61877 4.52571 3.50703 3.70318 3.96220 1.14679 3.46901 4.33439 2.69761 3.97839 2.85612 2.41443 2.94125 3.17874 3.61315 5.53323 4.27062	101 k - - -

2.68618 4.42225 2.77519 2.73123 3.46354 2.40513 3.72494 3.29354 2.67741 2.69355 4.24690 2.90347 2.73739 3.18146 2.89801 2.37887 2.77519 2.98518 4.58477 3.61503

0.02614 4.05309 4.77543 0.61958 0.77255 0.48576 0.95510

96	2.71907 5.14745 1.80450 1.91958 4.44617 3.33105 3.64820 3.90566 2.46601 3.43575 4.22934 2.81061 3.81101 2.78014 2.70790 2.67643 2.96630 3.17586 5.61050 4.20144	102 d - - -

2.68618 4.42225 2.77519 2.73123 3.46354 2.40513 3.72494 3.29354 2.67741 2.69355 4.24690 2.90347 2.73739 3.18146 2.89801 2.37887 2.77519 2.98518 4.58477 3.61503

0.02614 4.05309 4.77543 0.61958 0.77255 0.48576 0.95510

97	3.09546 4.76895 3.52647 3.23768 2.48253 3.78227 3.64023 3.40528 3.17478 2.93274 4.00712 2.91647 4.25474 3.51401 3.43795 3.20337 3.37355 3.19817 4.11038 1.08991	103 y - - -

2.68618 4.42225 2.77519 2.73123 3.46354 2.40513 3.72494 3.29354 2.67741 2.69355 4.24690 2.90347 2.73739 3.18146 2.89801 2.37887 2.77519 2.98518 4.58477 3.61503

0.02614 4.05309 4.77543 0.61958 0.77255 0.48576 0.95510

98	2.76062 4.77696 2.77659 2.34632 4.50168 1.03526 4.06878 3.96813 2.99240 3.60591 4.49910 3.12836 3.91215 3.30412 3.37908 2.83541 3.15368 3.54484 5.70826 4.46566	104 g - - -

2.68618 4.42225 2.77519 2.73123 3.46354 2.40513 3.72494 3.29354 2.67741 2.69355 4.24690 2.90347 2.73739 3.18146 2.89801 2.37887 2.77519 2.98518 4.58477 3.61503

0.02614 4.05309 4.77543 0.61958 0.77255 0.48576 0.95510

99	1.76554 4.23516 3.37984 3.19536 4.42705 1.18295 4.32872 3.80020 3.34095 3.53672 4.39097 3.35481 3.74857 3.61732 3.63659 2.47765 2.80313 3.24896 5.75554 4.56155	105 g - - -

2.68618 4.42225 2.77519 2.73123 3.46354 2.40513 3.72494 3.29354 2.67741 2.69355 4.24690 2.90347 2.73739 3.18146 2.89801 2.37887 2.77519 2.98518 4.58477 3.61503

0.02614 4.05309 4.77543 0.61958 0.77255 0.48576 0.95510

100	2.99855 5.58770 1.38863 1.45960 4.87230 3.24845 3.80555 4.39844 2.83838 3.89407 4.75266 2.70990 3.87817 2.97382 3.44450 2.87397 3.28990 3.96649 6.04183 4.52846	106 d - - -

2.68618 4.42225 2.77519 2.73123 3.46354 2.40513 3.72494 3.29354 2.67741 2.69355 4.24690 2.90347 2.73739 3.18146 2.89801 2.37887 2.77519 2.98518 4.58477 3.61503

0.02614 4.05309 4.77543 0.61958 0.77255 0.48576 0.95510

101	2.67662 4.79997 3.02732 2.39031 4.04828 3.46017 3.65213 3.43220 1.91248 2.39884 3.88901 3.01157 2.91061 2.81679 2.72191 2.72239 2.90444 3.13175 5.29281 3.99390	107 k - - -

2.68618 4.42225 2.77519 2.73123 3.46354 2.40513 3.72494 3.29354 2.67741 2.69355 4.24690 2.90347 2.73739 3.18146 2.89801 2.37887 2.77519 2.98518 4.58477 3.61503

0.02614 4.05309 4.77543 0.61958 0.77255 0.48576 0.95510

102	3.68755 4.91121 4.66810 4.36479 1.25459 4.42638 3.48677 3.36815 4.20852 2.69105 3.96224 4.10172 4.73300 4.16396 4.23266 3.79779 3.90868 3.29496 3.62210 1.13973	108 y - - -

2.68618 4.42225 2.77519 2.73123 3.46354 2.40513 3.72494 3.29354 2.67741 2.69355 4.24690 2.90347 2.73739 3.18146 2.89801 2.37887 2.77519 2.98518 4.58477 3.61503

0.02614 4.05309 4.77543 0.61958 0.77255 0.48576 0.95510

103	2.63974 4.46894 3.29040 2.27366 3.65192 3.55571 3.80536 2.44633 2.52709 2.64275 3.56804 3.21628 3.94789 3.05920 3.06726 2.81317 2.87462 1.98868 5.07922 3.81998	109 v - - -

2.68618 4.42225 2.77519 2.73123 3.46354 2.40513 3.72494 3.29354 2.67741 2.69355 4.24690 2.90347 2.73739 3.18146 2.89801 2.37887 2.77519 2.98518 4.58477 3.61503

0.02614 4.05309 4.77543 0.61958 0.77255 0.48576 0.95510

104	2.85786 5.37856 1.86308 2.08000 4.65276 3.28202 3.22938 4.16577 2.63917 3.66592 4.48110 1.70396 3.84227 2.86902 3.18566 2.77189 3.12395 3.75033 5.82108 4.35469	110 n - - -

2.68618 4.42225 2.77519 2.73123 3.46354 2.40513 3.72494 3.29354 2.67741 2.69355 4.24690 2.90347 2.73739 3.18146 2.89801 2.37887 2.77519 2.98518 4.58477 3.61503

0.02614 4.05309 4.77543 0.61958 0.77255 0.48576 0.95510

105	2.20331 4.42401 3.15310 2.86321 4.28088 1.34847 4.02413 3.69035 2.84788 3.34928 4.20030 3.19557 3.79593 3.25113 2.78638 2.57631 2.85987 3.23995 5.57102 4.31910	111 g - - -

2.68618 4.42225 2.77519 2.73123 3.46354 2.40513 3.72494 3.29354 2.67741 2.69355 4.24690 2.90347 2.73739 3.18146 2.89801 2.37887 2.77519 2.98518 4.58477 3.61503

0.02614 4.05309 4.77543 0.61958 0.77255 0.48576 0.95510

106	2.63144 4.74945 3.02915 2.00023 3.98146 3.44988 3.65460 3.31591 2.12744 2.97774 3.45869 3.00200 3.84727 2.82068 2.82723 2.68591 2.86187 2.49840 5.26568 3.95619	112 e - - -

2.68618 4.42225 2.77519 2.73123 3.46354 2.40513 3.72494 3.29354 2.67741 2.69355 4.24690 2.90347 2.73739 3.18146 2.89801 2.37887 2.77519 2.98518 4.58477 3.61503

0.02614 4.05309 4.77543 0.61958 0.77255 0.48576 0.95510

107	2.50826 4.34659 4.54853 4.03957 3.59356 4.18810 4.76668 1.24265 3.93723 2.21091 3.42736 4.26229 4.56631 4.20993 4.15755 3.55373 3.23050 1.45349 5.41897 4.21631	113 i - - -

2.68618 4.42225 2.77519 2.73123 3.46354 2.40513 3.72494 3.29354 2.67741 2.69355 4.24690 2.90347 2.73739 3.18146 2.89801 2.37887 2.77519 2.98518 4.58477 3.61503

0.02614 4.05309 4.77543 0.61958 0.77255 0.48576 0.95510

108	2.83033 4.30320 4.25409 3.72670 3.55293 3.97053 4.49226 1.73116 3.62661 2.28132 3.42589 3.98784 4.37726 3.91429 3.87908 3.31143 2.20273 1.38247 5.26470 4.05540	114 v - - -

2.68618 4.42225 2.77519 2.73123 3.46354 2.40513 3.72494 3.29354 2.67741 2.69355 4.24690 2.90347 2.73739 3.18146 2.89801 2.37887 2.77519 2.98518 4.58477 3.61503

0.02614 4.05309 4.77543 0.61958 0.77255 0.48576 0.95510

109	2.73987 4.90635 2.83031 2.47530 4.13224 3.40193 3.16500 3.66190 2.41619 3.23148 4.08133 2.97797 1.89533 2.43169 2.78851 2.76216 2.99484 3.32965 5.38079 4.02775	115 p - - -

2.68618 4.42225 2.77519 2.73123 3.46354 2.40513 3.72494 3.29354 2.67741 2.69355 4.24690 2.90347 2.73739 3.18146 2.89801 2.37887 2.77519 2.98518 4.58477 3.61503

0.01767 4.04462	* 0.61958 0.77255 0.00000	*

//
